# HucMSC-EVs Facilitate *In Vitro* Development of Maternally Aged Preantral Follicles and Oocytes

**DOI:** 10.1007/s12015-022-10495-w

**Published:** 2023-03-02

**Authors:** Ying-Yi Zhang, Weijie Yang, Yi Zhang, Zhanhong Hu, Yingyan Chen, Yerong Ma, Anran Yang, Zhan Shi, Hanjing Zhou, Peipei Ren, Libing Shi, Jiamin Jin, Yan Rong, Xiaomei Tong, Yin-Li Zhang, Songying Zhang

**Affiliations:** 1 Assisted Reproduction Unit, Department of Obstetrics and Gynecology, Sir Run Run Shaw Hospital, School of Medicine, Zhejiang University, Hangzhou, China; 2 Department of Obstetrics and Gynecology, Key Laboratory of Reproductive Dysfunction Management of Zhejiang Province, Hangzhou, China

**Keywords:** Human umbilical cord mesenchymal stem cells, Extracellular vesicles, Follicle development, Oocyte maturation, Female fertility, Transcription, Advanced maternal age

## Abstract

Follicle developmental capacity and oocyte quality decline with advanced maternal age. Extracellular vesicles from human umbilical cord mesenchymal stem cells (HucMSC-EVs) act as a potential therapeutic product in the treatment of age-related ovarian dysfunction. In vitro culture (IVC) of preantral follicles is a useful method for understanding the mechanism of follicle development and is a promising means for improving female fertility. However, whether HucMSC-EVs have beneficial effects on aged follicle development during IVC has not yet been reported. Our research demonstrated that follicular development with single-addition withdrawal of HucMSC-EVs was better than that with continuous treatment with HucMSC-EVs. HucMSC-EVs facilitated the survival and growth of follicles, promoted the proliferation of granulosa cells (GCs), and improved the steroid hormone secretion of GCs during IVC of aged follicles. Both GCs and oocytes could uptake HucMSC-EVs. Moreover, we observed elevated cellular transcription in GCs and oocytes after treatment with HucMSC-EVs. The RNA sequencing (RNA-seq) results further validated that the differentially expressed genes are related to the promotion of GC proliferation, cell communication, and oocyte spindle organization. Additionally, the aged oocytes displayed a higher maturation rate, presented less aberrant spindle morphology, and expressed a higher level of the antioxidant protein Sirtuin 1 (SIRT1) after treatment with HucMSC-EVs. Our findings suggested that HucMSC-EVs can improve the growth and quality of aged follicles and oocytes in vitro through the regulation of gene transcription, which provides evidence for HucMSC-EVs as potential therapeutic reagents to restore female fertility with advanced age.

**Graphical Abstract:**

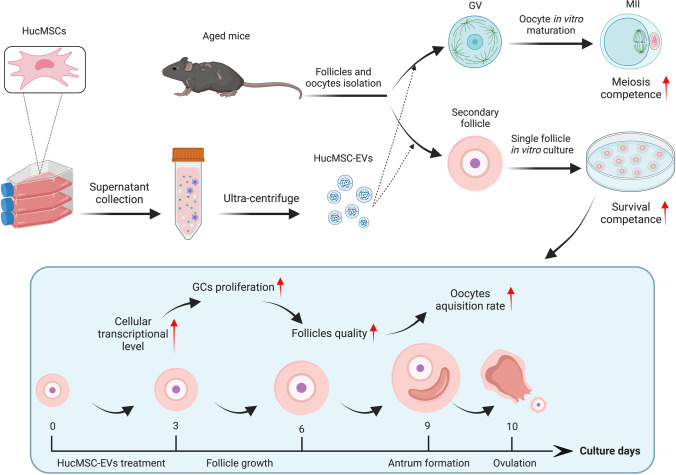

**Supplementary Information:**

The online version contains supplementary material available at 10.1007/s12015-022-10495-w.

## Background

Ageing has become a global concern due to its heavy impact on health, and organ function declines with age. In females, reproductive ageing occurs prior to that of other systems, and age-related female reproductive deficiency is rarely reversible [1]. Ovary is a crucial reproductive organ that secrets hormones as part of its endocrine function and produces fertilization-competent oocytes [2]. The follicle serves as a basic unit in the ovary and is composed of an oocyte surrounded by many GCs and theca cells. The number of follicles gradually decreases over time with cyclic recruitment [3]. Many studies have demonstrated that aged ovarian follicles have a lower capacity for growth due to imbalanced oxidant defence systems and increased apoptosis among somatic cells [4, 5]. Moreover, oocyte quality declines with advanced maternal age, thus resulting in a lower capacity for meiotic maturation and further fertilization or a higher likelihood of adverse pregnancy outcomes [6]. Despite the importance of improving follicle growth and oocyte quality to restore aged female fertility, efficient strategies have not yet been proposed.

In recent decades, mesenchymal stem cells (MSCs) have emerged as a novel therapeutic product for a variety of diseases. To date, MSCs have been proven to mitigate the age-related decline in ovarian reserve [7] and improve the reproductive function of patients and animal models with premature ovarian insufficiency (POI) disease [8] by in vivo administration. In addition, studies have reported that MSCs play a positive role in treating diseases, which is partially accomplished through MSC-derived extracellular vesicles (EVs). EVs are believed to participate in communication between donor cells and receptor cells by delivering proteins, miRNAs, and lncRNAs, among other molecules [9]. Compared with the functions of MSCs, the biological functions of EVs are more stable, but the immunogenicity of EVs as a type of cell-free regenerative medicine is lower than that of MSCs [10]. Among the various types of MSCs, human umbilical cord-derived mesenchymal stem cells (HucMSCs) have been shown to be easily obtainable and possess great potential for therapeutic effects on female reproductive disease in previous studies [11].

It is well-recognized that the number of follicles at all stages decreases during advanced maternal ageing [12]. In recent years, in vitro activation (IVA) of primordial follicles has emerged as a potential therapy for patients with POI or diminished ovarian reserve (DOR) in aged females [13]. Despite the high rate of activation of folliculogenesis from the dormant stage, not all of the activated follicles can favourably develop into healthy and dominant follicles. Some researchers believe that the system of combining IVA of primordial follicles with IVC of growing follicles (IVA-IVC) to acquire mature oocytes may eliminate repeated surgery and minimize the carcinogenic risk for patients [14]. In addition, it has been proposed that not only the primordial follicles but the preantral follicle pool are potentially valuable resources for fertility preservation [15]; additionally, human ovarian biopsies can provide a viable source of preantral follicles [16]. Follicle IVC systems have been established for mice, humans, and many other mammalian species [17], and they can be used to obtain mature oocytes. Researchers have demonstrated the potential effects of MSCs on the promotion of preantral follicle growth during IVC [18, 19].

As is currently known, follicles from aged mice cultured in vitro have been confirmed to abnormally secrete hormones, as well as contain low-quality oocytes that have decreased meiotic competence and extrude metaphase II (MII) eggs with chromosome alignment defects upon acquisition [20]. However, the majority of stem-cell/ extracellular-vesicle therapies aimed at the promotion of follicular rescue were focused on IVA [21], whereas less attention was paid to the later improvement of preantral follicles in patients with impaired or aged ovarian reserves. Therefore, further optimization is needed to improve the IVC system of preantral follicles for obtaining useful mature oocytes, in order for it to become an encouraging strategy for POI/DOR patients and females of advanced maternal age who want to restore fecundity and achieve successful pregnancy.

HucMSC-EVs have been proven to have constructive effects on ovarian function and fertility preservation [22], whereas studies that concentrate on improving the compacity and quality of aged follicles have not been conducted. Moreover, it is difficult to study the effect of HucMSC-EVs on follicle growth and oocyte quality and to characterize the underlying mechanism in vivo; the IVC system is an ideal approach for studying the effects of HucMSCs or HucMSC-EVs on ovarian follicles. In this study, we hypothesized that HucMSC-EVs may be a potential therapeutic reagent to treat the age-related decline in follicle developmental deficiency in vitro. Specifically, we conducted a coculture system of HucMSC-EVs and preantral follicles from aged mice to demonstrate that HucMSC-EVs facilitate the growth and quality of follicles and the maturation of oocytes. Moreover, we found that the possible mechanism of follicle growth promotion was through increases in cellular transcriptional levels. Our study is the first to demonstrate the positive function of HucMSC-EVs in aged follicles and oocytes, and our findings may help in the construction of a potential ovarian follicle culture system, as well as provide a new perspective on age-related ovarian fertility preservation.

## Materials and Methods

### Experimental Animals

All of the mice were obtained from the Animal Center of Sir Run Run Shaw Hospital and housed in a specific pathogen-free (SPF) environment of 23–25 °C with a 12/12-hour light/dark cycle and 40–70% humidity. The study was conducted with the approval of the Committee on the Ethics of Animal Experiments at Sir Run Run Shaw Hospital, Zhejiang University. The sample size was not predetermined by specific statistical methods. In all of the experiments, young mice included approximately 2-3-week-old C57BL/6 female mice, and aged mice referred to approximately 8-10-month-old C57BL/6 female mice. VASA-RFP transgenic mice (RBRC03449, RIKEN, Japan) with red fluorescence in oocytes were used to label the oocytes and more clearly observe the distribution of EVs after coculture.

### Isolation, Culture, and Identification of HucMSCs

Fresh umbilical cords were collected from women with full-term healthy pregnancies who underwent caesarean sections with informed consent at the Sir Run Run Shaw Hospital, School of Medicine, Zhejiang University. The tissues were washed twice with saline containing 100 U/mL penicillin, 100 mg/mL streptomycin, and 250 ng/mL amphotericin B (triple antibiotics, Solarbio, China), and the umbilical vein and both umbilical arteries were removed. The remaining tissues were cut into 1-mm^3^ to 5-mm^3^ pieces, plated in sterile dishes with 8 mL complete culture medium composed of DMEM/F-12 (Meilunbio, China), 10% foetal bovine serum (FBS, Cellmax, China), and 1% triple antibiotics, and cultured in an incubator at 37℃ with 5% CO_2_ for 10–14 days. The culture medium was changed every other day. After the cell density reached approximately 70–80% confluence, the cells were digested with 0.25% trypsin-EDTA solution (Solarbio, China).

At passage 5, the phenotype markers of HucMSCs were evaluated via flow cytometry analysis by using the Human Mesenchymal Stem Cell Detection Kit (HUXMX-09011, Cyagen, China), with positive markers CD29, CD44, CD73, and CD166 and negative markers CD45, CD11b, CD34, CD14, and human leukocyte antigen (HLA)-DR. Flow cytometry analysis was performed following relevant instructions (Beckman Coulter, USA). The osteogenic, chondrogenic, and adipogenic differentiation experiments of HucMSCs were performed by specific culture systems (HUXUC-90,021, HUXUC-90,041, and HUXUC-90,031, Cyagen, USA, respectively), and the differentiated cells were stained with alcian blue, alizarin red, and oil red O, respectively.

### Isolation and Identification of HucMSC-EVs

HucMSC-EVs were isolated from the supernatant of HucMSCs at passages 5 to 9. Complete culture medium was removed when cells reached a density of 60–70% in the dishes, and cells were washed twice with phosphate-buffered saline (PBS, Gibco, USA) and then replaced with conditioned culture medium containing 10% EVs-free FBS and 1% triple antibiotics. In EVs-free FBS, EVs were deprived from the FBS via ultracentrifugation (L-100XP, Beckman Coulter, USA) at 105,000×g for 16 h. After 48 h of culture, the supernatant was collected and centrifuged at 800×g for 10 min to deplete dead cells, 2000×g for 20 min to remove cell debris and 10,000×g for 30 min to eliminate large extracellular vesicles. After filtration through 0.22-µm disposable syringe filters (Millipore, USA), the supernatant was ultracentrifuged at 105,000×g for 70 min to isolate small-sized extracellular vesicles, which were named exosomes [23, 24]. The precipitate was then suspended in cool PBS and ultracentrifuged at 105,000×g for 70 min to remove contaminating proteins. All centrifugations and ultracentrifugations were performed at 4 °C. Finally, the pellets were resuspended in 200 ~ 300 µL precooled sterile PBS and frozen at -80 °C for future use. The concentrations of the solutions at the protein level were measured by Qubit 4.0 (Invitrogen, USA).

Experiments on the characterization and identification of EVs were conducted according to a published protocol [23]. Particle size distribution and concentrations were measured by nanoparticle tracking analysis (NTA, Particle Metrix, Germany). EVs were fixed with 2.5% glutaraldehyde overnight at 4 °C, and their morphologies were observed by using transmission electron microscopy (TEM, Tecnai G2 Spirit BioTWIN, Thermo Fisher, USA) in the Center of Cryo-Electron Microscopy, Zhejiang University. The positive markers of EVs, including exosomal surface markers of ALG-2-interacting protein X (ALIX), Tumor Susceptibility Gene 101 (TSG101), Glyceraldehyde-3-Phosphate Dehydrogenase (GAPDH), tetraspanins CD63 and CD81, and negative markers of EVs (including intracellular protein 94 kDa glucose-regulated protein [GRP94]), were detected via western blot analysis.

### Protein Preparation and LC‒MS/MS Analysis

Three HucMSC-EVs samples from independent experiments were collected and prepared as protein samples. Briefly, the protein concentration was measured by Qubit 4.0. A total of 200 µg of protein solution was used and digested with sequencing-grade trypsin (Promega, Madison, USA). Then, a C18 Cartridge was used to desalt the digested peptide. Liquid chromatography-tandem MS (LC‒MS/MS) analysis was subsequently conducted on a Q-Exactive mass spectrometer (Thermo Fisher, USA) coupled with an Easy-nLC liquid chromatography system (Thermo Fisher, USA) for 120 min in Applied Protein Technology (APTBIO, Shanghai, China) according to a previously published protocol [25]. Maxquant software (version 1.5.3.17) and the SwissProt Human proteome database containing 20,395 proteins (SwissProt_Homo_sapiens_20395_20210106.fasta) were utilized to analyse the raw data. The proteins identified by the database search met the set filter parameter of a false discovery rate (FDR) ≤ 0.01. The identified proteins were compared with available extracellular vesicle data from the ExoCarta database (http://www.exocarta.org). Both Gene Ontology (GO) and Kyoto Encyclopedia of Genes and Genomes (KEGG) term analyses were performed using the DAVID online tool (https://david.ncifcrf.gov).

### Isolation and *In Vitro* Culture of Early Secondary Preantral Follicles

Young and aged mice were sacrificed with carbon dioxide. Then, the ovaries were removed and placed in M2 medium (M7167, Sigma-Aldrich, USA). Healthy early secondary preantral follicles with diameters of 85–135 μm contained an intact and round oocyte in the central, several layers of granulosa cells and a visible theca cell layer, were mechanically isolated and separated carefully from ovaries by using insulin syringes. Follicles were then individually cultured in a single droplet containing 10 µL follicle IVC medium covered by mineral oil (Sigma-Aldrich, USA) in a sterile dish (Corning, USA). Follicle IVC medium was composed of alpha minimum essential medium (α-MEM, Gibco, USA) supplemented with 0.1 U/mL follicle-stimulating hormone (FSH, Lizhu, China), 3 mg/mL bovine serum albumin (BSA, Sigma-Aldrich, USA), 5% FBS (Cellmax, China), 1% triple antibiotics, 0.23 mM pyruvic acid (Sigma-Aldrich, USA), 50 µg/mL ascorbic acid (Sigma-Aldrich, USA), 5 mg/mL insulin, 5 mg/mL transferrin and 5 ng/mL selenium (ITS, Sigma-Aldrich, USA). The follicles were cultured at 37 °C with 5% CO_2_ and 100% humidity in an incubator. The culture medium was refreshed every three days.

Images of follicles were captured every two or three days using an inverted microscope (Eclipse TE200, Nikon, Japan). Follicle diameter (µm) measurements were conducted by ImageJ software (version 1.8.0.112), measuring the distance between two sides of the follicle.

After 10 days of culture, the in vitro ovulation and luteinization experiments were executed on antral follicles characterized by the formation of a fluid-filled antrum. For induction of ovulation, the culture medium was replaced by a maturation medium supplemented with 1.5 IU/ml human chorionic gonadotrophin (hCG, American Pharmaceutical Partners, USA) and 5 ng/ml recombinant human epidermal growth factor (rEGF, Novus, USA). After 16 h of ovulation induction, cumulus-oocyte complexes (COCs) were imaged and countered, medium samples were collected for hormone assays, and GCs were collected into lysis buffer for gene expression analysis.

### Oocyte Collection and *In Vitro* Maturation

For IVM studies, aged and young mice were intraperitoneally injected with 10 IU and 5 IU of pregnant mare serum gonadotropin (PMSG, Sansheng, China), respectively. After 44–48 h, the mice were humanely sacrificed. Oocytes at the germinal vesicle (GV) stage were harvested by using mechanical isolation and cultured in preequilibrated M2 medium with or without 5 µg/mL (7.3 × 10^8^ particles/mL) HucMSC-EVs under mineral oil at 37 °C with 5% CO_2_ for 16 h. The matured oocytes at the MII stage (with visible polar bodies) were countered and selected for future studies.

For the acquisition of in vivo ovulated (IVO) MII oocytes, each mouse was intraperitoneally injected with human chorionic gonadotropin (hCG, Sansheng, China) at the same dose as PMSG at 44–48 h post-PMSG, after which they were euthanized after 14–16 h. The MII oocytes and cumulus cell complex were collected from the ampulla of fallopian tube in the M2 medium with 3 mg/ml hyaluronidase (H4272, Sigma-Aldrich).

### Uptake of HucMSC-EVs by Follicles and Oocytes

Follicles and oocytes were obtained from 3-week-old female VASA-RFP transgenic mice. EVs were suspended with PKH67 green fluorescent dye (Sigma-Aldrich, USA) according to the manufacturer’s instructions for 4 min at room temperature and then blocked with 1% BSA/PBS solution to avoid excessive staining. Centrifugal filter devices (Millipore, USA) were applied to eliminate the residual dye. Labelled EVs were then added to the medium for follicle culture and oocyte culture. After incubation in the dark for approximately 10 h, follicles and oocytes were collected, stained with Hoechst 33,342 and observed by confocal microscopy.

### Western Blot Analysis

The western blot analysis procedure was published in previous studies [26]. Briefly, proteins were extracted with RIPA lysis buffer (R0010, Solarbio, China) containing a protease inhibitor cocktail (P8340, Sigma-Aldrich, United States), and the protein concentrations were measured by using the Qubit 4.0. Subsequently, the proteins were diluted with Laemmli protein sample buffer (1,610,747, Bio-Rad, USA). After denaturation for 10 min at 95 °C, equal amounts of extracted proteins (around 10 µg each) were separated via electrophoresis on 10% sodium dodecyl sulphate-polyacrylamide gel (SDS-PAGE, PG112, Epizyme, China) and transferred to polyvinylidene difluoride (PVDF) membranes (Millipore, USA). The membranes were blocked in TBST buffer containing 5% skimmed milk for at least 1 h and then incubated overnight at 4 °C with primary antibodies. The detected proteins related to the primary antibodies that were used in the study were listed in Supplementary Table 4. After washing with TBST buffer three times, the membranes were incubated with secondary antibodies and visualized by enhanced chemiluminescence (WBKLS0500, Millipore, USA) using a ChemiDoc Touch imaging system (Bio-Rad, USA).

### Gene Expression Analysis

Gene expression was analysed by using real-time quantitative polymerase chain reaction (qPCR). Oocytes and GCs of follicles were separated mechanically, collected, and lysed in lysis buffer. Lysis buffer in a total of 4 µL contained 2 µL 0.2% Triton X-100 with 2 IU/µL RNase inhibitor, 1 µL 10 mM dNTP mix, and 1 µL 10 µM oligo-dT primer as described previously [27]. According to the procedure of Smart-seq2 [28], we performed cDNA synthesis and amplification for qPCR templates by using SuperScript II (18,064,014, Invitrogen, USA) and KAPA HiFi HotStart ReadyMix (KK2602, Roche, Switzerland), respectively. The qPCR procedure was performed according to the SYBR Green Mix protocol (Vazyme, China) on a CFX-Connect platform (BioRad, USA). The transcriptional levels of genes were normalized to *Actin* and compared with the levels of these genes in the control group. The primer sequences of the targeted genes are listed in Supplementary Table 1.

### Hormone Assays

The supernatants of follicle culture medium were assembled and stored at -80 °C for hormone assays, and every 15 to 25 droplets (150 µL to 250 µL) were aggregated as one sample. The 17β-estradiol and progesterone levels in the supernatants were measured with Access Immunoassay System (UniCel DxI 800, Beckman Courter, USA) by the clinical laboratory department of Sir Run Run Shaw Hospital, School of Medicine, Zhejiang University.

### Immunofluorescence and Confocal Detection

Oocytes, follicles, or fresh ovary tissue sections were fixed in 3.7% paraformaldehyde (PFA) for 30 min at room temperature and then washed with 0.1% BSA/PBS solution three times. After being permeabilized with 0.2% Triton X-100 (Sigma-Aldrich, USA) for 30 min and then washed three times with PBS, oocytes, follicles, or tissue sections were blocked in blocking solution (1% BSA/PBS with 0.1% Triton X-100) for 1 h, followed by incubation with primary antibodies overnight at 4 °C. After washing in 0.1% BSA/PBS three times, oocytes or follicles were incubated with the secondary antibody while nuclei were counterstained with Hoechst. Both primary and secondary antibodies were diluted in blocking solution. After 45 min of incubation at room temperature, oocytes or follicles were placed on slides using an anti-fade reagent (Beyotime, China) and observed by confocal microscopy (Zeiss LSM800, Zeiss, Germany). Semiquantitative analysis of the fluorescence intensity was performed using ZEN software (version 2012) matched to the confocal microscope.

### Detection of Transcription by EU Incorporation Assay

Follicles cultured with or without HucMSC-EVs after 3 days were collected and cultured in IVC medium with 100 µM 5’-ethynyl uridine (EU), which is a uracil nucleoside analogue that can infiltrate synthetic RNA molecules with uracil (U) during RNA transcription, for another 2 h. According to the manufacturer’s protocol of the Click-iT RNA Alexa Fluor 488 Imaging kit (C10329, Thermo Fisher, USA), follicle fixation, permeabilization, blocking, and EU sinaling detection were performed, with subsequent incubation with RNA Polymerase II CTD repeat YSPTSPS (phospho S2) (PSII) antibody overnight in the dark. Secondary antibody incubation and image acquisition of follicles were conducted as above.

### RNA Sequencing (RNA-seq) and Data Analysis

Similar-sized follicles cultured after 3 days in the two groups were collected, and oocytes and GCs of follicles were separated mechanically. Five growing oocytes were lysed in 4 µL lysis buffer as one sample, 0.1 µL of ERCC (External RNA Controls Consortium) spike-in RNA (4,456,740, Invitrogen, USA) was added as the external control, and cDNA was acquired as above using the Smart-seq2 method. Sequencing libraries were constructed by Annoroad Gene Technology Corporation (Beijing, China) with KAPA KK8514 (Roche, Switzerland) according to the manufacturer’s protocol. Samples were sequenced by Illumina HiSeq 2500 for 125-bp paired-end sequencing. All the raw reads were filtered, and clean reads in FASTQ format were obtained. The reads were then mapped against the mouse reference genome (mm10) by using STAR (version 2.5.2a). The mapped reads were subsequently assembled into transcripts with featureCounts (version 2.0.1). The expression levels of genes were determined by fragments per kilobase of transcripts per million mapped reads (FPKM) and calibrated by ERCC [29] according to the instructions of the product. FPKM should be over 1 at least in one sample in each gene. In addition, DESeq2 [30] was used to analyse the transcripts in the original data and assess differentially expressed genes (DEGs) at a *P* value of < 0.05 and Log_2_FC (fold change) of > 0.5. GO and KEGG analyses were performed as mentioned above. Scatter plots, heatmaps of the selected genes, and relative GO and KEGG terms were drawn by using the online tool (https://www.bioinformatics.com.cn), a free platform for data analysis and visualization. Principal component analysis (PCA) and a heatmap of all DEGs were generated to show the clustering of all samples.

### Statistical Analysis

GraphPad 9.0 and SPSS 26.0 software were used for all statistical analyses. All quantitative results are shown as the means ± SEMs. Each measurement was performed at least three independent experiments. Differences between two groups were evaluated by two-tailed unpaired Student’s *t*-tests, while for comparisons of more than two groups, one-way ANOVA was used to evaluate differences. *P* < 0.05 indicated statistical significance.

## Results

### Identification and Characteristics of HucMSCs and HucMSC-EVs

**Fig. 1:**
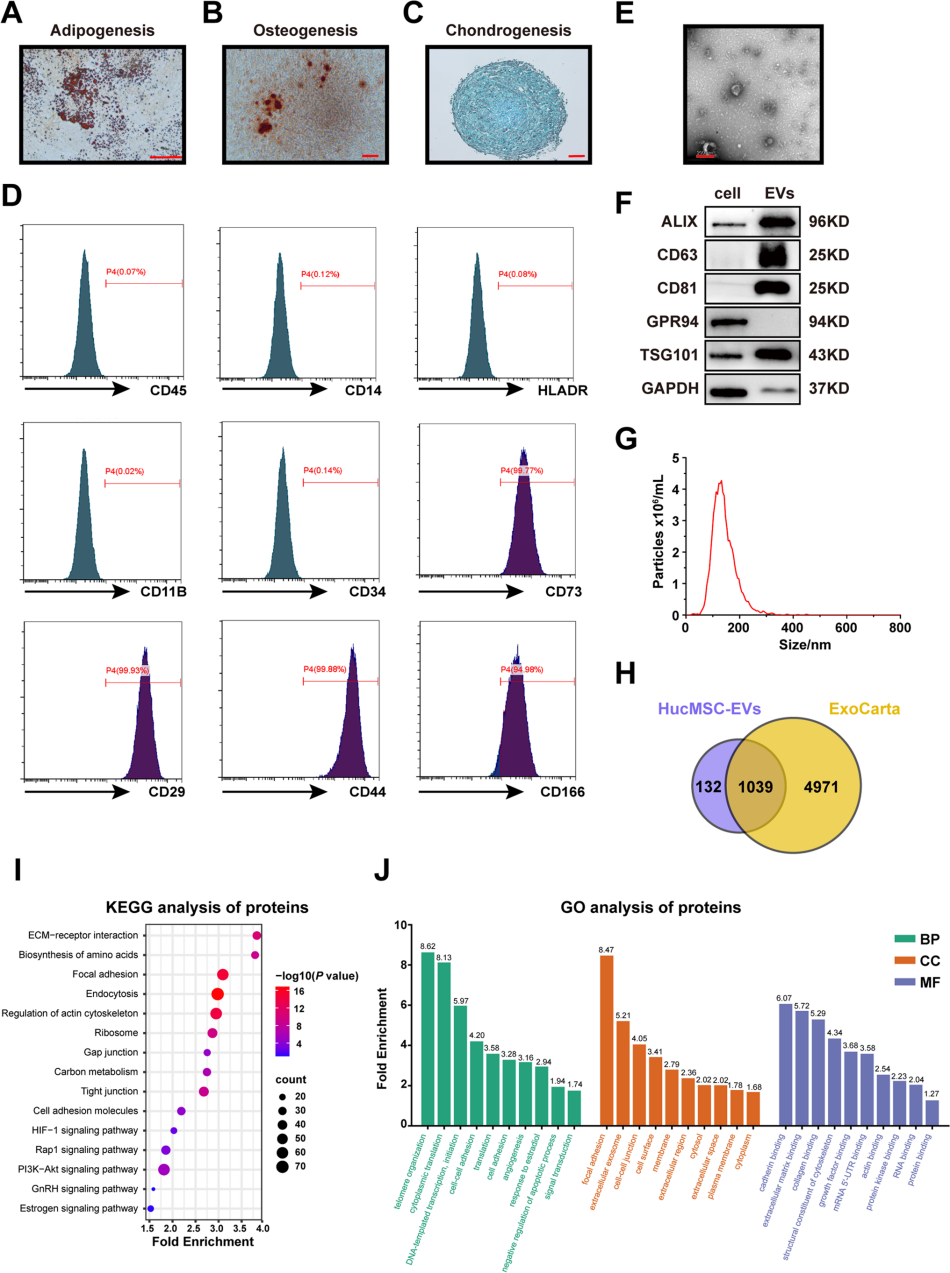
Characteristics of HucMSCs and HucMSC-EVs. Representative images of **A** adipocytes (oil red), **B** osteoblasts (alizarin red), and **C** chondrocytes (alcian blue) in standard in vitro differentiation medium. Scale bar = 25 μm. **D** The surface markers of HucMSCs, including negative markers (CD45, CD14, HLA-DR, CD11B, and CD34) and positive markers (CD73, CD29, CD44, and CD166), were detected by flow cytometry. **E** The morphology of HucMSC-EVs was observed by using TEM. Scale bar = 200 nm. **F** The protein expression levels of ALIX, CD63, CD81, GRP94, TSG101, and GAPDH in the HucMSC-EVs were compared with those in the HucMSCs via western blot. **G** Size distributions of HucMSC-EVs were detected by using NTA. **H** Venn diagram of the proteins in the HucMSC-EVs against ExoCarta. **I-J** Bioinformatics analysis of the proteomics of HucMSC-EVs. **I** The KEGG pathway was enriched with the protein in HucMSC-EVs. **J** Relative GO terms of the protein in HucMSC-EVs were obtained, and fold enrichment was labelled on each term

The primary generation of HucMSCs was obtained from umbilical cords after approximately 10–14 days of culture. At passage five, we observed cells showing classic fibroblast-like morphology. Then, we performed multilineage differentiation potential experiments in conditional culture systems followed by protocols. HucMSCs were successfully differentiated into adipocytes with lipid droplets stained by oil red O, osteoblasts with bone matrix stained by alizarin red, and chondroblasts stained by alcian blue (Fig. 1A-C). Flow cytometry showed that HucMSCs expressed the positive markers CD29, CD44, CD73, and CD166 (but not the negative markers CD11b, CD34, CD45, CD14, and human leukocyte antigen [HLA]-DR) (Fig. 1D).

We collected EVs from the conditioned medium of HucMSCs. These EVs met the minimal experimental criteria for the definition of extracellular vesicles and their functions [23]. Specifically, HucMSC-EVs were characterized by their typical cup-shaped morphology under TEM (Fig. 1E). Western blot analysis demonstrated the presence of exosomal surface markers of ALIX, TSG101, CD63, and CD81, as well as the absence of intracellular proteins GRP94 (Fig. 1F). NTA showed that the size distribution of these particles ranged from approximately 100 to 180 nm (Fig. 1G), and the original particle concentration was approximately 7.3 × 10^10^ particles/mL. The protein concentration of these EVs was approximately 0.5 µg/µL, as measured by Qubit 4.0.

Moreover, we performed proteomic analysis of HucMSC-EVs by using LC-MS/MS to detect potential proteins and possible functional mechanisms. A detailed proteomic analysis in three independent samples revealed a total of 1172 different proteins (Supplementary Table 2), 88.65% (1039) of which could be matched in ExoCarta (Fig. 1H), known as a conventional database of EVs. KEGG pathway enrichment and GO analysis demonstrated several pathways related to the functions of HucMSC-EVs (Fig. 1I-J), which is similar to previously reported results [31]. Taken together, these results indicated that the EVs that were purified in this study were specific and of high quality.

### Effects of HucMSC-EVs on Preantral Ovarian Follicles from Young Mice

**Fig. 2:**
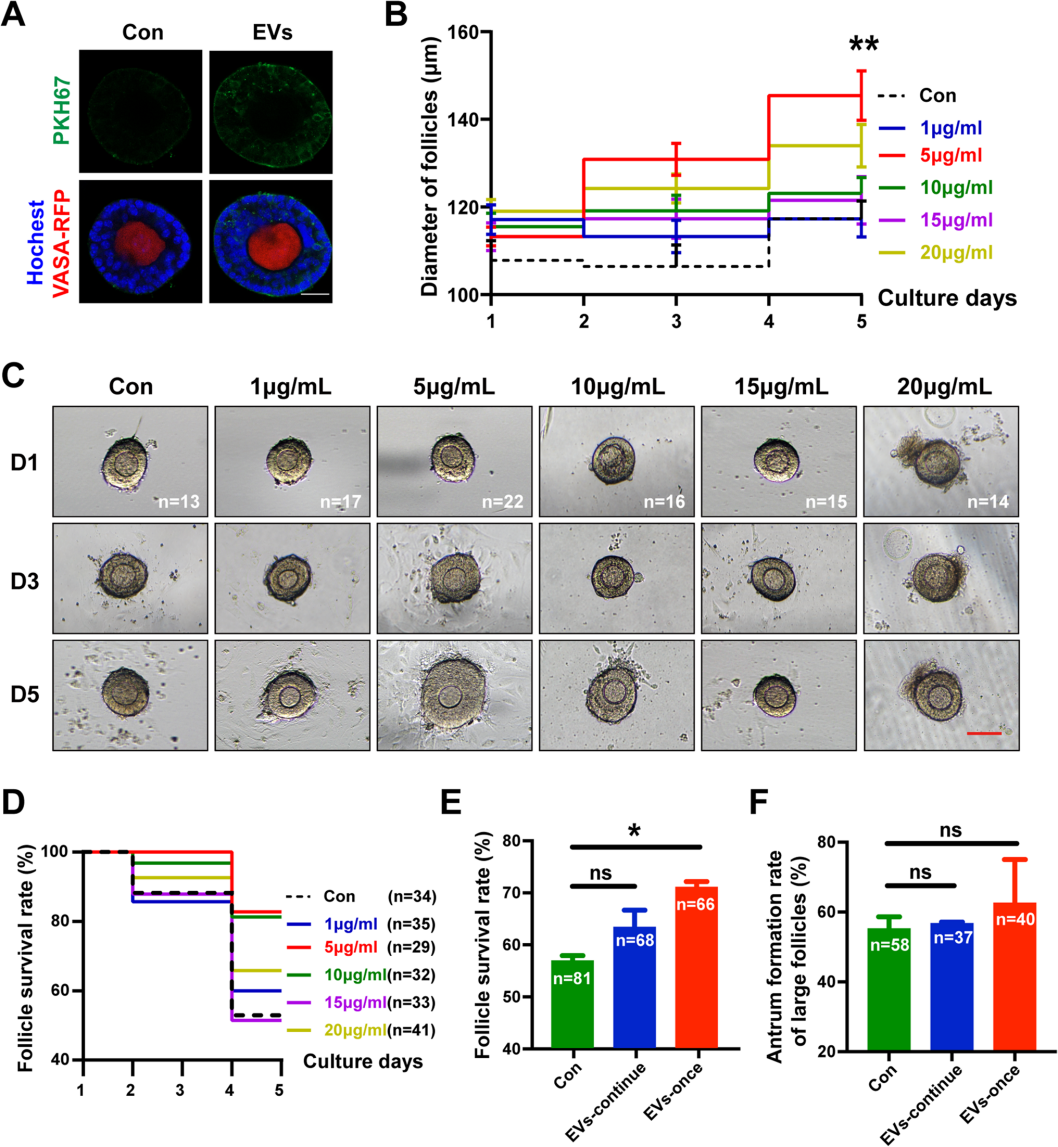
Effects of HucMSC-EVs on murine follicle culture *in vitro*. **A** Uptake of HucMSC-EVs by cultured preantral follicles. Nuclei were stained with Hoechst 33,342. Green indicates EVs labelled with PKH67. Red indicates oocytes containing the VASA protein. **B** The diameters of the follicles in the 6 groups were measured. **C** Representative morphologies of follicles cultured in different dosages of HucMSC-EVs for 5 days. Scale bar = 100 μm. **D** Follicle survival rate of groups at different dosages. **E** Follicle survival rate and **F** antrum formation of the three groups; con refers to the control group without HucMSC-EVs, EVs-continue refers to the follicles cocultured with HucMSC-EVs throughout the IVC time, and EVs-once refers to the follicles cocultured with HucMSC-EVs for 3 days. The concentrations of 1, 5, 10, 15, or 20 µg/mL refer to 1.5 × 10^8^, 7.3 × 10^8^, 1.5 × 10^9^, 2.2 × 10^9^, and 2.9 × 10^9^ particles/mL of HucMSC-EVs, respectively. *P < 0.05, **P < 0.01

To observe whether the HucMSC-EVs could be efficiently taken up by mouse preantral follicles, we used VASA-RFP transgenic mice, in which the oocytes showed red fluorescence and PKH67 dye with green fluorescence to label the membranes of the HucMSC-EVs. As shown in Fig. 2A, after coculture with labelled HucMSC-EVs for approximately 10 h, follicles in the experimental group exhibited the ability to uptake HucMSC-EVs, and most were taken up into GCs whereas others were taken up into oocytes.

To determine the best conditions for supplementation, we used 1, 5, 10, 15, or 20 µg/mL (1.5 × 10^8^, 7.3 × 10^8^, 1.5 × 10^9^, 2.2 × 10^9^, or 2.9 × 10^9^ particles/mL, respectively) concentrations of HucMSC-EVs in coculture with small-sized preantral follicles (< 120 μm) from young mice. To evaluate the growth of follicles in these groups, follicle diameters were measured on day 1, day 3, and day 5 of culture. We found no significant differences among the groups on day 1 or day 3. However, follicles in the 5 µg/mL group showed larger diameters (145.43 ± 5.67 μm, n = 22) than the control group (117.30 ± 4.08 μm, n = 13) (*P* < 0.01), 1 µg/mL group (117.26 ± 4.13 μm, n = 17), 10 µg/mL group (123.12 ± 3.58 μm, n = 16), 15 µg/mL group (121.54 ± 5.43 μm, n = 15), and 20 µg/mL group (133.99 ± 4.88 μm, n = 14) (Fig. 2B). Surviving follicles were identified by an intact oocyte in a regular shape surrounded by several layers of GCs (Fig. 2C). In addition, on day 5, the 5 µg/mL group had a higher percentage of living follicles (82.76%, n = 29) than the control (52.94%, n = 34), 1 µg/mL (60.00%, n = 35), 10 µg/mL (81.25%, n = 32), 15 µg/mL (51.52%, n = 33) and 20 µg/mL (65.85%, n = 41) groups (Fig. 2D). These results indicated that 5 µg/mL HucMSC-EVs had a better effect on murine follicle growth.

Thus, 5 µg/mL (7.3 × 10^8^ particles/mL) HucMSC-EVs was determined to be the best concentration in the follicle IVC medium. To evaluate whether the temporary or permanent effect of EVs on follicles would perform better, we replaced the medium with fresh IVC medium on the third day. We withdrew HucMSC-EVs, replaced them with fresh IVC medium without HucMSC-EVs on the EVs-once group follicles, and maintained them by using 5 µg/mL HucMSC-EVs on the EVs-continue group follicles. On day 7 of in vitro growth, the survival rate of the EVs-once group (71.11 ± 1.11%, n = 66) was higher than that of the control group (*P* < 0.05) and the EVs-continue group (56.85 ± 1.04%, n = 81 and 63.33 ± 3.34%, n = 68, respectively) (Fig. 2E). The antrum formation rate of the large follicles in the EVs-once group (62.5 ± 12.5%, n = 40) was also higher than that in the control group or EVs-continue group (55.17 ± 3.45%, n = 58 and 56.70 ± 0.45%, n = 37, respectively), although the difference was not significant (*P* > 0.05) (Fig. 2F). These results suggested that follicle coculture with 5 µg/mL HucMSC-EVs for 3 days was the method for optimal function in further experiments.

### HucMSC-EVs Facilitate the Growth of Aged Preantral Follicles *In Vitro*

**Fig. 3:**
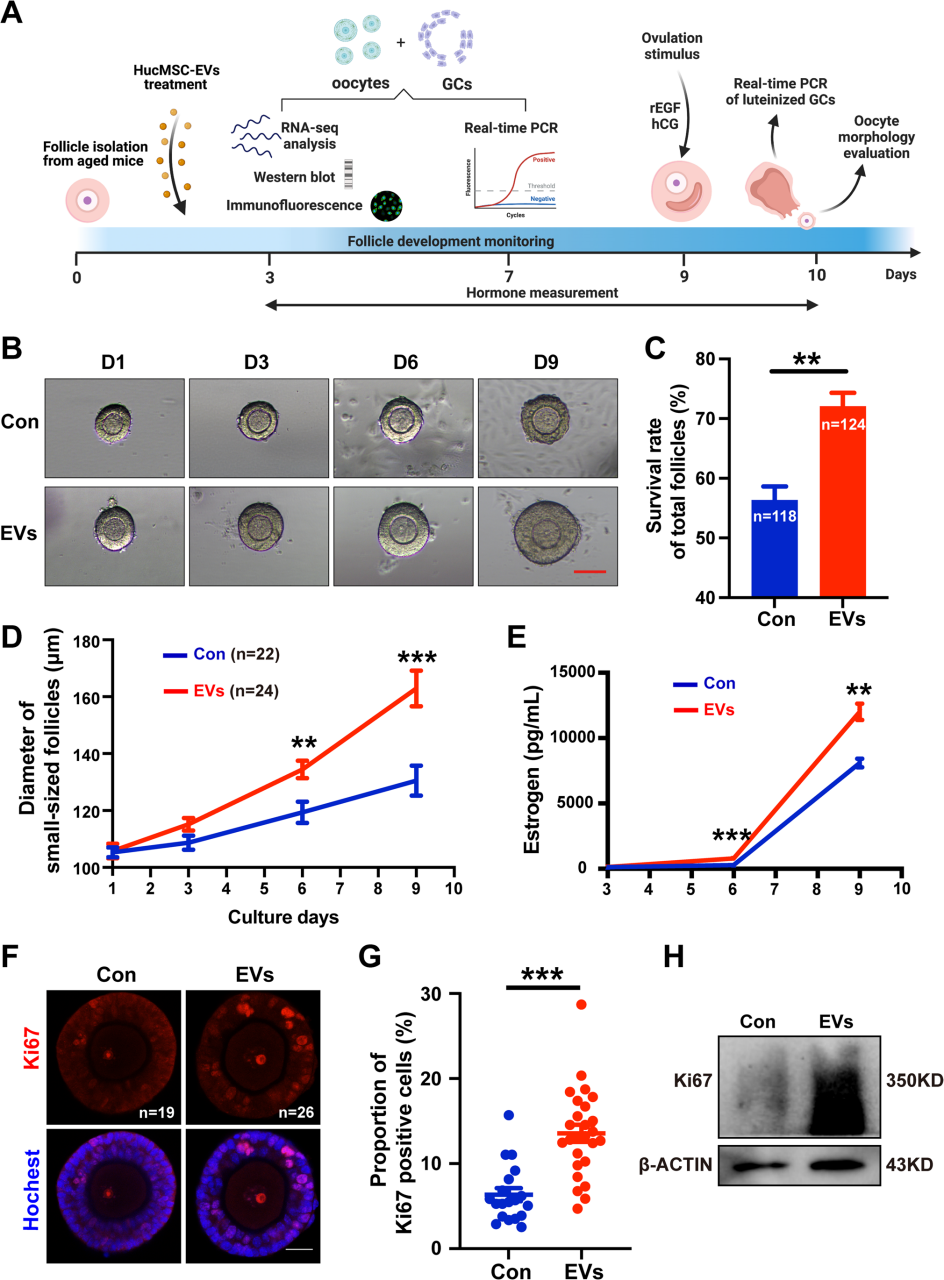
HucMSC-EVs improve the survival and growth of aged follicles during IVC. **A** A schematic diagram showing the experimental design and measurements during IVC of aged follicles. **B** Representative photomicrographs of aged follicle development in vitro with or without HucMSC-EVs. Scale bar = 100 μm. **C** Quantitative analysis of the survival rate among all of the follicles with (n = 124) or without (n = 118) HucMSC-EVs. **D** Follicle diameters were measured in small-sized follicles with (n = 22) or without (n = 24) HucMSC-EVs. **E** Estrogen levels secreted from follicles were tested in two groups during follicle growth (n = 4–6). **F** Immunofluorescence of Ki67 in secondary follicles treated with (n = 26) or without (n = 19) HucMSC-EVs. Red represents Ki67. Blue represents Hoechst 33,342. Scale bar = 25 μm. **G** Analysis of the proportion of Ki67-positive GCs in the largest layer of the follicle by using ImageJ. **H** Ki67 expression level in follicles detected by western blot. *P < 0.05, **P < 0.01, ***P < 0.001

Numerous researchers have believed that approximately 8-10-month-old female mice could mimic physiological reproductive aging in approximately 35-40-year-old women [32, 33]; therefore, we chose 8-10-month-old female murine ovaries to isolate aged preantral follicles as the targets. Preantral follicles were isolated from aged mice, cocultured with HucMSC-EVs for three days, and then replaced with fresh culture medium for another 6 days of culture, followed by ovulation induction. At the indicated time points, some follicles were collected for different experiments, as shown in Fig. 3A. Different initial sizes of the follicles would cause divergences on the same culture conditions for the same culture days [34]; therefore, it is important to control the primary size of the follicles to precisely demonstrate the effect of HucMSC-EVs on aged follicles. Early preantral follicles from aged mice were divided into two groups: follicles whose diameters were below 120 μm were divided into the small-size group, and follicles above 120 μm were deemed large-size follicles. In both groups of follicles, HucMSC-EVs were able to improve the overall size and survival rate (72.02 ± 2.31%, n = 124) compared to the control group (56.32 ± 2.30%, n = 118) (*P* < 0.01) (Fig. 3B-C).

Among the survived small-sized follicles, the initial diameters of the group with HucMSC-EVs and the control group were 105.75 ± 12.09 μm (n = 22) and 105.28 ± 8.73 μm (n = 24), respectively (Fig. 3D). The follicle diameters gradually increased in both groups, but they increase faster in the experimental group than in the control group (Fig. 3B and D). On day 3, the difference between the EVs group (115.18 ± 10.49 μm) and the control group (108.67 ± 12.22 μm) was not significant (Fig. 3D). However, on day 6, the diameters of the EVs group reached 134.44 ± 14.43 μm, which was larger than that of the control group at 119.39 ± 18.57 μm (*P* < 0.01). On day 9 of culture, divergence was more evident (*P* < 0.001). Follicles cocultured with HucMSC-EVs reached a size of 162.89 ± 29.35 μm, whereas those follicles in the control group were only 130.52 ± 25.76 μm (Fig. 3D).

17β-estradiol is mainly secreted by ovarian follicles, and a certain level of estrogen (E2) is considered the characteristic feature of follicle growth [35]. The E2 level was remarkably increased during follicle IVC, and HucMSC-EVs were observed to be promoters toward follicles on E2 production (Fig. 3E). On day 6, follicles with HucMSC-EVs produced a much higher level of E2 in culture medium (784.36 ± 76.81 pg/mL, n = 6) than that in the control group (276.69 ± 30.89 pg/mL, n = 6) (*P* < 0.001). Similarly, on day 9, the E2 level of the EVs group (12011.60 ± 629.04 pg/mL, n = 4) was higher than that of the control group (8087.96 ± 337.08 pg/mL, n = 4) (*P* < 0.01).

To validate the positive impact on the growth potential of aged follicles from HucMSC-EVs, follicle immunofluorescence was performed on day 3 to detect the protein expression of Ki67, which is a biomarker associated with cell proliferation [36]. The proportion of Ki67-positive GCs in the largest layer of an individual follicle was increased in the EVs group (13.49 ± 1.01%, n = 26) compared with the control group (6.25 ± 0.78%, n = 19) (*P* < 0.001) (Fig. 3F-G). Consistent with the immunofluorescence results, we found that Ki67 protein expression was also upregulated in the aged follicles treated with HucMSC-EVs by western blot analysis (Fig. 3H). Collectively, these results indicated that HucMSC-EVs facilitate the survival and growth of aged preantral follicles in vitro.

To explore the potential mechanism of HucMSC-EVs promoting the development of aged follicles, we performed western blot analysis on small-sized and large-sized aged preantral follicles at 4 h, 8 h, 16 h, and 24 h after coculture respectively. We found rather than the upregulation of the mTOR/rpS6 signaling pathway which serves as one of the downstream pathways of phosphatidylinositol 3-kinase (PI3K)/protein kinase B (Akt) and is highly related to cellular survival, growth, and proliferation [37], a temporary activation of cAMP response element (CRE)-binding protein (CREB)/ KIT ligand (KITL) /PI3K/Akt signaling at 8 h timepoint in both sizes of follicles (Supplementary Fig. 2A-B), which is close to the time period of uptake HucMSC-EVs by follicles. The activation sustained longer until 16 h in large-sized follicles, which might be due to the larger amount of particles of follicles uptake. However, the activation in follicles returned to baseline after 24 h, which might be the reason why we previously discovered that the aged preantral follicles which experienced continuous exposure to HucMSC-EVs did not develop better than the once-treatment group (Fig. 2E-F). In addition, we found downregulation of Histone deacetylase 6 (HDAC6) expression in the granulosa cells (Supplementary Fig. 2C-D) after 8 h of coculture with HucMSC-EVs and found an obvious increase of SIRT1 in the EVs group at 8 and 16 h-time-points, while there seemed no significant change between the two groups after 24 h of coculture (Supplementary Fig. 2E), which might be the potential activation regulators of PI3K/Akt signaling [38, 39].

### HucMSC-EVs Facilitate the Ovulation Response of Aged Follicles *In Vitro*

The proliferation performance of preantral follicles labelled by Ki-67 in young and aged ovaries showed no apparent differences (Supplementary Fig. 1A), and the total survival rate also showed no divergences between young (63.08 ± 4.05%, n = 78) and aged follicles (61.47 ± 3.32%, n = 80) (*P* > 0.05) (Supplementary Fig. 1B), thus indicating that the process of follicular size expansion was not strictly restricted by age. Additionally, it was similar to the results mentioned in the previous studies, wherein the follicles from aged mice contained oocytes with reduced meiotic competence and increased spindle defects, whereas the diameters of follicles did not have significant divergence, which reflected the different follicular function (but comparable growth speed) between young and aged follicles [20]. Furthermore, some aged follicles exhibited similar gene expression signatures compared to the young follicles by RNA-seq analysis of single follicles, thus indicating the considerable variation of the aged follicles. Such heterogeneity could also be observed in humans, wherein not all of the aged females would experience the same difficulty in fertilization and pregnancy [40].

**Fig. 4:**
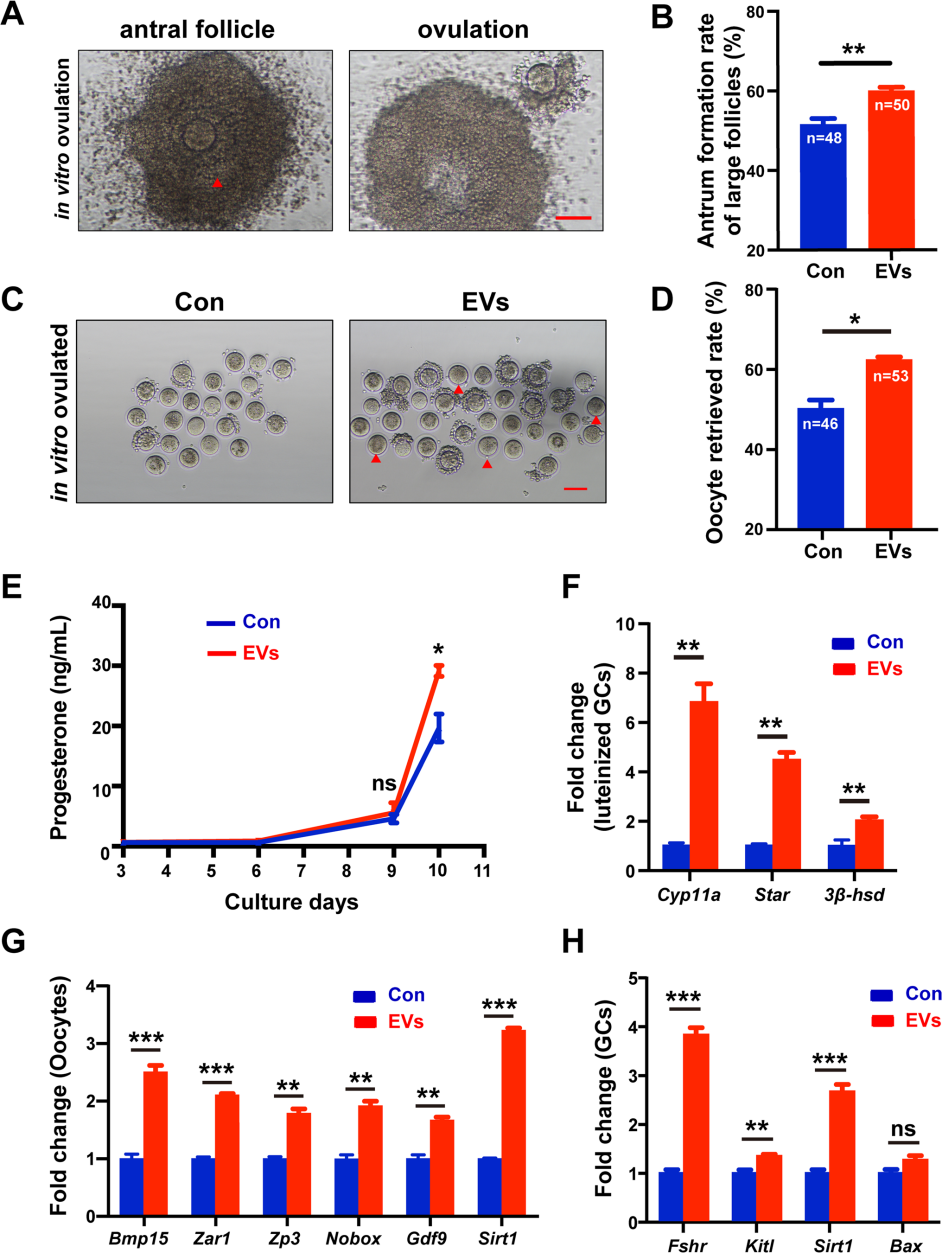
HucMSC-EVs facilitate the ovulation response and improve the quality of aged follicles during IVC.** (A** Representative images of antral follicles and oocytes after ovulation stimulation. The red arrowhead indicates the antrum cavity. Scale bar = 100 μm. **B** The antrum formation rate of large-sized survived follicles with (n = 50) or without (n = 48) HucMSC-EVs was assessed. **C** Phase-contrast images of oocytes ovulated from aged follicles after ovulation stimulation. The red arrowhead indicates the oocytes that reached GVBD. Scale bar = 100 μm. **D** The oocyte retrieval rate of the total survived follicles was assessed in the EVs group (n = 53) and the control group (n = 46). **E** Progesterone levels secreted by follicles during follicle development and after hCG and EGF stimulation (n = 3–4). **F** qPCR analysis of relative luteinization gene mRNA levels in follicular GCs after ovulation stimulus. qPCR analysis of relative mRNA levels in **G** oocytes and **H** GCs separated mechanically from aged follicles on culture day 7. *P < 0.05, **P < 0.01, ***P < 0.001

The antrum formation rate of large-sized survived follicles was assessed on day 9. The follicle diameters of the antral follicles were usually above 300 μm, and the antrum cavity is indicated by an arrowhead (Fig. 4A, left panel). The antrum formation rate of large-sized follicles was considerably decreased in the aged group (56.82 ± 9.64%, n = 27) compared to the young group (73.21 ± 1.79%, n = 26), in spite of a lack of significance (*P* = 0.146) (Supplementary Fig. 1C). When being supplemented with HucMSC-EVs, the antrum formation rate was significantly increased to 59.96 ± 0.93% (n = 50) compared to the control group (51.52 ± 1.52%, n = 48) (*P* < 0.01) (Fig. 4B). After a maturation stimulus by hCG and rEGF for 16 h on day 10, oocytes ovulated by the antral follicles were collected and counted (Fig. 4A, right panel). The oocyte retrieved rate varied considerably between the young (85.80 ± 1.70%, n = 84) and aged follicles (50.00 ± 2.17%, n = 46) (*P* < 0.01) (Supplementary Fig. 1E). Inconsistent with the aged group (wherein no healthy mature oocytes were found), oocytes ovulated from young follicles exhibited higher meiotic competence, with several follicles at the MII stage (Supplementary Fig. 1D), which directly suggested the higher quality of preantral follicles from young mice. However, the oocyte retrieved rate was elevated greatly in the EVs group (62.25 ± 0.71%, n = 53) (Fig. 4C-D) (*P* < 0.05), which suggested the rescue effect of HucMSC-EVs on impaired aged follicles. The abovementioned results proved that HucMSC-EVs could facilitate the growth of both small- and large-sized aged preantral follicles in vitro and subsequently improve oocyte maturation capacity.

Due to the fact that follicle luteinization after ovulation to maintain the serum progesterone (P4) level and further influence embryo implantation in vivo is of considerable importance [41], the concentration of P4 in the follicle supernatant was measured. Ovulation stimulus was conducted by hCG and rEGF to obtain mature oocytes and evaluate the process of follicle luteinization. There was no significant difference in the P4 level between the control group and the EVs group during the 9 days of culture (n = 3–4) (*P* > 0.05). However, after 16 h of the ovulation stimulus, the level of P4 in the EVs group (29.16 ± 0.92 ng/mL, n = 4) was notably higher than that in the control group (19.67 ± 2.32 ng/mL, n = 4) (*P* < 0.05) (Fig. 4E). Then, the luteinized GCs after ovulation were collected to detect the expression of genes related to steroidogenesis and follicle luteinization, which included cholesterol side-chain cleavage cytochrome P450 (*Cyp11a1*), steroidogenic acute regulatory protein (*Star*), and 3β-hydroxysteroid dehydrogenase (*Hsd3b1*). In our study, HucMSC-EVs upregulated the expression of these genes (Fig. 4F) (*P* < 0.01). Therefore, HucMSC-EVs were confirmed to support the luteinization process of aged follicles in vitro.

### HucMSC-EVs Promote the Development of Aged Follicles by Upregulating the Cellular Transcriptional Level

We performed qPCR to determine the gene expression analysis of separated growing oocytes and GCs from aged follicles after 7 days of culture. Similar sizes of at least 3 follicles were selected for one sample. The messenger RNA (mRNA) expression levels of genes related to follicle growth and oocyte quality were elevated in the EVs group in both oocytes, including bone morphogenetic protein 15 (*Bmp15*), zygote arrest-1 (*Zar1*), zona pellucida-3 (*Zp3*), newborn ovary homeobox gene *(Nobox*), growth differentiation factor 9 (*Gdf9*), and *Sirt1* and in GCs, including follicle-stimulating hormone receptor (*Fshr*), kit ligand (*Kitl*), and sirtuin 1 (*Sirt1*) (*P* < 0.01) (Fig. 4G-H). This result demonstrated that HucMSC-EVs improved the overall quality of aged preantral follicles during IVC at the molecular level.

**Fig. 5:**
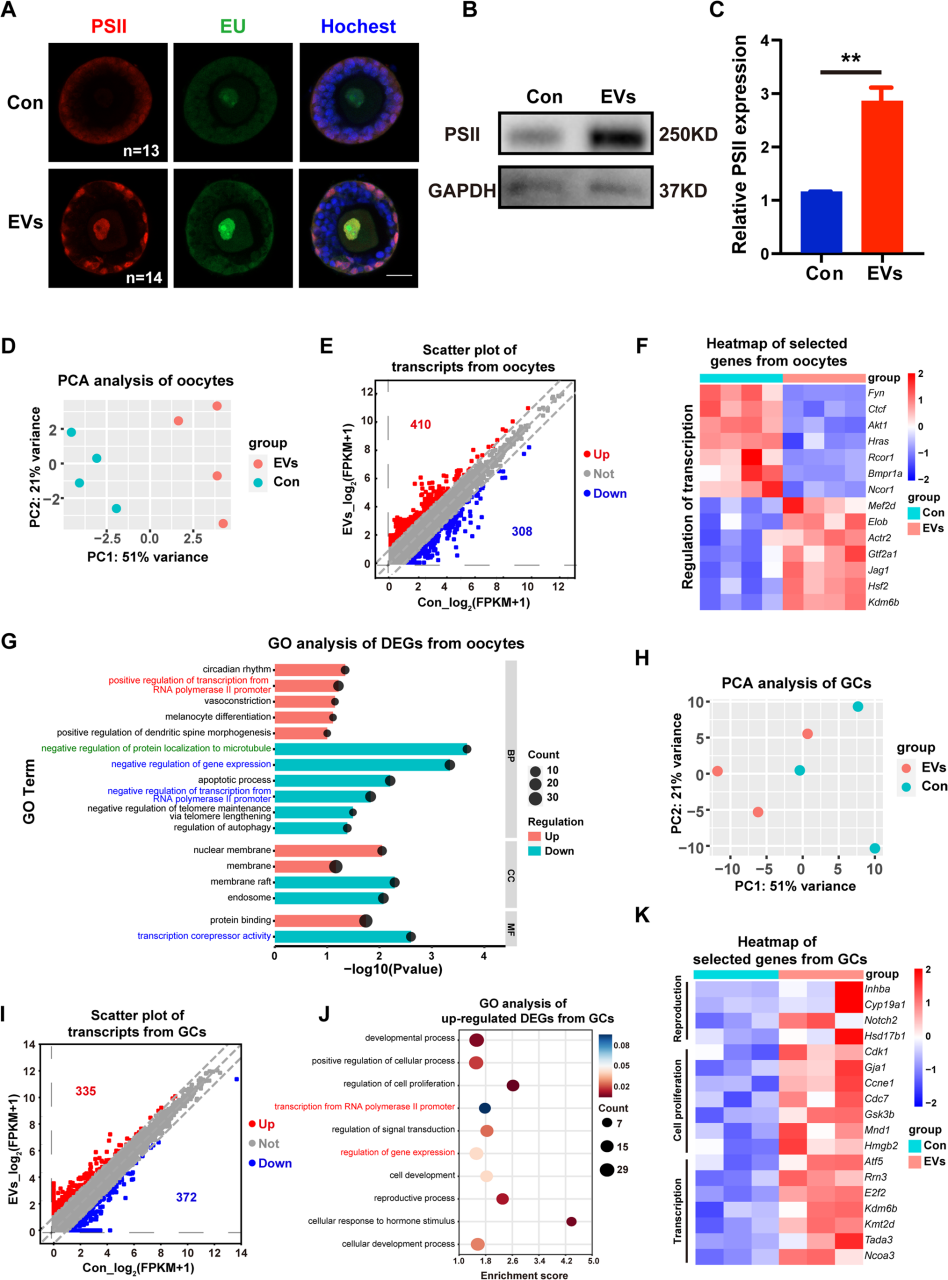
HucMSC-EVs improved the global cellular transcription level of aged follicles. **A** Immunostaining images of cellular transcription-related markers of follicles in the two groups (n = 13–14); red represents PSII, green represents EU, and blue represents cell nuclei stained with Hoechst 33,342. **B** The PSII expression level was detected by western blot analysis, and GAPDH was used as the internal control. **C** Quantitative measurement of relative PSII expression was shown by using ImageJ. **D-G** RNA-seq profiling of oocytes isolated from aged follicles with or without HucMSC-EVs treatment. **D** Principal component analysis (PCA) diagram showing the distinguishing clusters of eight oocyte samples from the control and the EVs group. **E** Scatter plot presenting oocyte transcriptional divergence between the two groups by FPKM levels. Red spots indicate to upregulated genes, and blue indicates downregulated genes. Log2 (fold change) > 1. **F** Heatmap showing differences in cellular transcription activity-related genes in oocytes from the two groups. **G** GO enrichment of oocyte DEGs between the two groups was analysed; BP, biological process; CC, cellular component; and MF, molecular function. **H-K** RNA-seq profiling of GCs isolated from aged follicles. **H** PCA, **I** scatter plot of transcriptional divergence and **J** GO enrichment of upregulated DEGs were performed. **K** Heatmap of selected genes in GCs related to reproduction, cell proliferation and transcription initiation

During folliculogenesis, the volume of human oocytes increases approximately 100-fold [42], accompanied by high-quality maternally derived mRNA and necessary protein accumulation for subsequent fertilization and embryonic development [43]. Based on this knowledge and our results (Fig. 4G-H), we postulated whether HucMSC-EVs promoted gene expression in growing follicles. The EU incorporation assay was employed to evaluate the global transcription level in aged follicles after culture for 3 days, together with the PSII protein level, an indicator of transcription activity [44]. We found that both EU and PSII signals were significantly increased in growing oocytes within the preantral follicles (Fig. 5A), thus indicating active transcription in oocytes after treatment with HucMSC-EVs. Some somatic cells were also demonstrated to have a high expression level of PSII in the EVs group, which is consistent with the observation via western blot analysis (Fig. 5B-C). Therefore, we observed an elevation in cellular transcriptional levels in follicles after HucMSC-EVs treatment.

To further investigate the possible mechanisms of the positive effect of HucMSC-EVs on aged follicles, we performed RNA-seq analysis of oocytes and GCs separated from aged follicles in the two groups. After 3 days of IVC, eight samples containing 5 growing oocytes each from similar-sized follicles were collected to perform the RNA-seq analysis. The transcriptomic profiles of four replicates in the two groups were determined to be comparable by using PCA (Fig. 5D), thus demonstrating the divergence in the oocytes between the control and EVs groups. As shown in the scatter plot, a total of 13,256 transcripts were detected. After treatment with HucMSC-EVs, 410 transcripts were upregulated, and 308 transcripts were downregulated (Fig. 5E). We selected several genes responsible for the regulation of cellular transcription (*Fyn*, *Bmpr1a*, *Hras*, *Kdm6b*, etc.) and compared their expression levels by FPKM, as shown in Fig. 5F. Then, we performed DESeq2 on the raw data to select the DEGs with *P* values < 0.05 and log2 (fold change) > 0.5. Heatmap clustering of all DEGs showed discrepant clustering between the two groups (Supplementary Fig. 3A). DEGs were subjected to Gene Ontology (GO) analysis in the categories of biological process (BP), cellular component (CC), and molecular function (MF). The enriched GO terms of the DEGs were predominantly related to transcriptional activation (Fig. 5G). Specifically, positive regulation of transcription was enriched among the upregulated genes (labelled in red), while negative regulation of transcription and gene expression, together with transcription corepressor activity, were enriched among the downregulated genes (labelled in blue). Otherwise, we found that the apoptotic process, the regulation of autophagy, and the negative regulation of telomere maintenance, which were highly associated with the age-related decline in oocyte quality [6], were enriched among the downregulated genes in the EVs group.

Similarly, we performed RNA-seq analysis on six samples of remaining GCs separated from follicles. Despite the existence of two close samples, PCA demonstrated the overall divergence between the two groups (Fig. 5H). A total of 15,624 transcripts were detected, among which 335 were upregulated and 372 were downregulated (Fig. 5I). GO term enrichment of upregulated DEGs after DESeq2 analysis also showed an elevated transcriptional level labelled in red (Fig. 5J). Moreover, genes related to the developmental process, reproductive process, and regulation of cell proliferation were upregulated in the EVs group, which is consistent with the results shown in Fig. 3. Specifically, some transcripts related to reproduction (*Inhba*, *Cyp19a1*, *Notch2*, *Hsd17b1*), cell proliferation (*Cdk1*, *Gja1*, *Ccne1*, *Cdc7*, etc.), and transcriptional activation (*Atf5*, *Kdm6b*, *Kmt2d*, *Tada3*, etc.) were selected to elucidate the difference in GCs between the two groups (Fig. 5K).

### HucMSC-EVs Improve the Meiotic Process of Aged Oocytes During *In Vitro* Maturation

Analogous to the results of human follicle culture in vitro [45], most of the ovulated oocytes in our study from the IVC system remained in the GV stage (Fig. 4A). Four oocytes in the EVs group reached GVBD, but none of the oocytes succeeded in maturing to the MII stage. Oocyte maturation is important for further fertilization and embryo development. We found that the oocytes obtained from aged mice after in vivo ovulation (IVO) decreased in both quantity and quality (Supplementary Fig. 1F). The number of ovulated oocytes per mouse were obviously different between the aged (26.25 ± 1.38, n = 3) and young (38.335 ± 2.03, n = 4) groups (*P* < 0.01) (Supplementary Fig. 1G). Among the total oocytes after IVO, the rate of abnormal morphology (including fragments, shrinkage, and degeneration) was significantly elevated in the aged group (28.87 ± 2.20%, n = 105) compared to the young group (6.12 ± 0.93%, n = 115) (*P* < 0.001) (Supplementary Fig. 1H). In addition to the divergences of oocyte meiosis in vivo, the in vitro maturation rate of aged GV oocytes was also obviously reduced (58.70 ± 2.41%, n = 108) in contrast to the young oocytes (76.30 ± 1.62%, n = 118) (*P* < 0.01) (Supplementary Fig. 1K-L), which is consistent with the decline of meiotic competence of in vitro matured human oocytes in higher donor ages [46], thus reflecting the follicle and oocyte development deficiency in ageing ovaries.

**Fig. 6:**
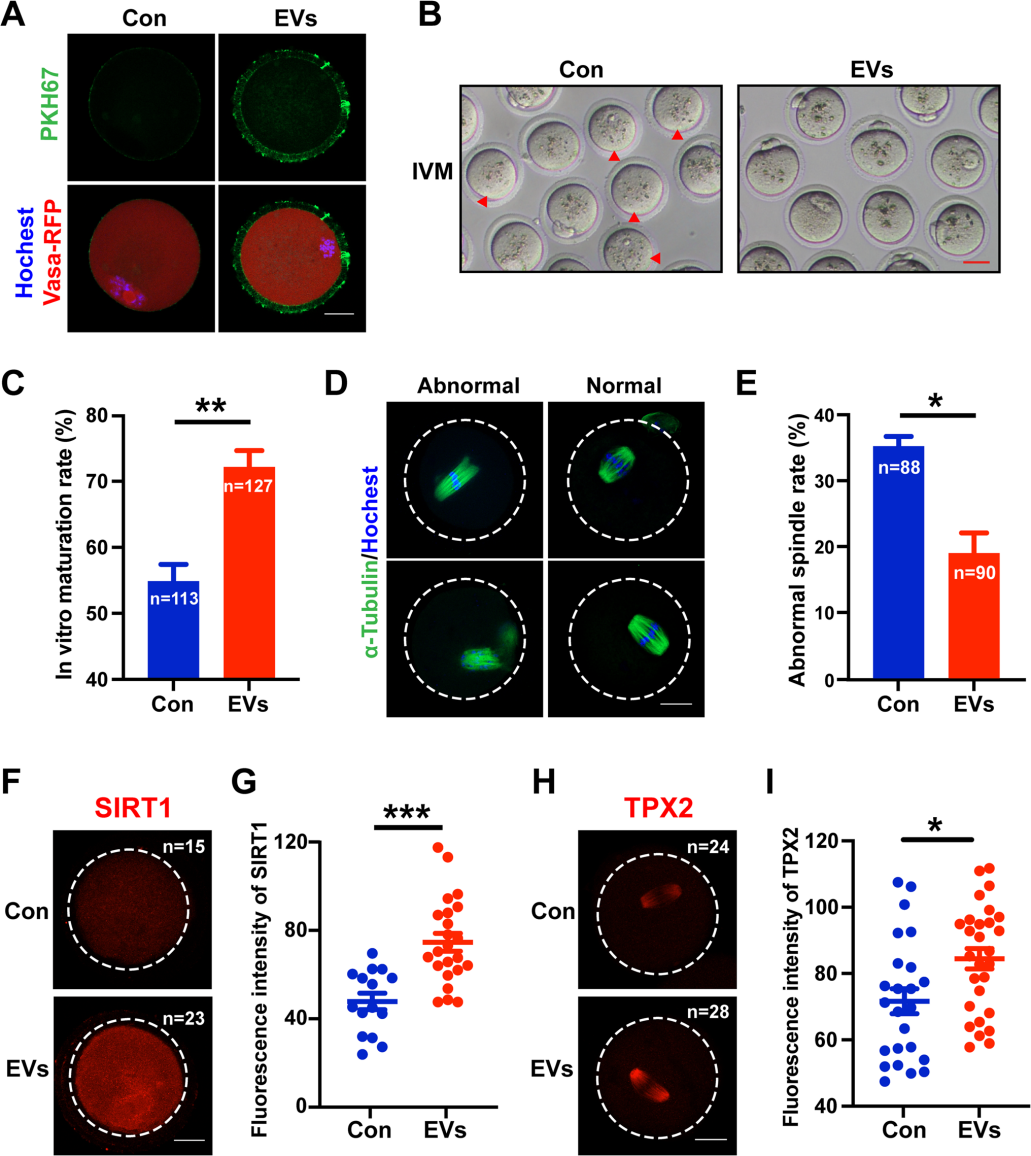
Effects of HucMSC-EVs on aged oocyte maturation *in vitro*. **A** Uptake of HucMSC-EVs by cultured oocytes. Nuclei were stained with Hoechst 33,342. Green indicates EVs labelled with PKH67. Red indicates oocytes. Scale bar = 25 μm. **B** Representative phase-contrast images of oocyte maturation in vitro with or without HucMSC-EVs. Scale bar = 50 μm. The red arrowhead indicates the oocytes that fail to extrude a polar body. **C** Quantitative measurement of the IVM rate in aged oocytes with (n = 127) or without (n = 113) HucMSC-EVs. **D** Representative images of normal and abnormal spindle organization in oocytes at the MII stage. Scale bar = 25 μm. Blue fluorescence represents nuclei with Hoechst 33,342. Green fluorescence indicates α-Tubulin-stained spindle morphology. **E** Quantification of oocytes with aberrant spindle assembly in the control (n = 88) and EVs groups (n = 90). **F** Representative confocal images of SIRT1 in the control and EVs groups. Scale bar = 25 μm. **G** Statistical analysis of the fluorescence intensity of SIRT1 in matured oocytes with (n = 15) or without HucMSC-EVs (n = 23). **H** Representative images of the cellular translation-related marker TPX2 in the two groups. Scale bar = 25 μm. **I** Statistical analysis of TPX2 fluorescence intensity in matured oocytes with (n = 28) or without HucMSC-EVs (n = 24). *P < 0.05, **P < 0.01, ***P < 0.001

To explore the potential effect of HucMSC-EVs on aged oocytes, in this study, we used fully grown oocytes in the GV stage from aged mice for IVM. The results showed that HucMSC-EVs can be taken up by both follicles and isolated oocytes in vitro (Fig. 6A). After coculture for 16 h in IVM medium, we found that the number of immature aged oocytes (without polar bodies) diminished in the EVs group (Fig. 6B). The proportion of MII-stage oocytes in the EVs group was significantly increased (72.00 ± 2.53%, n = 127) compared with that in the control group (54.74 ± 2.62%, n = 113) (*P* < 0.01) (Fig. 6C).

Then, we detected spindle morphology and antioxidant protein (SIRT1) levels to estimate the quality of the matured oocytes. We found a decreased protein expression level in the aged MII oocytes via immunofluorescence (Supplementary Fig. 1I-J). And aged oocytes usually display aberrant spindle morphologies with fragments, fracture, and segregation [47] (Fig. 6D). After treatment with HucMSC-EVs, the rate of abnormal spindles was lower (18.94 ± 3.13%, n = 90) than that in the control group (35.18 ± 2.65%, n = 88) (*P* < 0.05) (Fig. 6E). We also found that the SIRT1 expression level was higher in the EVs group than in the control group (Fig. 6F), and the difference was evident (*P* < 0.001) (Fig. 6G). Microtubule nucleation factor TPX2 (targeting factor for *Xenopus* kinesin-like protein 2) is known to function in spindle assembly in cells and regulate the meiotic maturation of oocytes [48]. Moreover, TPX2 protein expression is highly regulated by translation during oocyte maturation; thus, TPX2 protein levels were detected with or without treatment with HucMSC-EVs. As shown in Fig. 6H, TPX2 protein was significantly accumulated on the spindles of oocytes from the EV-treated group, thus indicating that coculture with HucMSC-EVs can enhance the mRNA translation level and improve spindle assembly capacity in aged oocytes during the meiotic maturation process (*P* < 0.05) (Fig. 6I), which was surprisingly consistent with the result that genes related to “negative regulation of protein localization to microtubules” were downregulated in the oocytes from the RNA-seq analysis (Fig. 5G, labelled in green).

## Discussions

It is well confirmed that ovarian function decline accelerates after 35 years of age in women, and age-associated female fertility preservation is of great concern in clinical practice [49]. Females with diminished ovarian reserve are usually advised to receive oocyte donation due to the rarity of spontaneous ovulation. In our previous studies and other studies, HucMSCs transplantation was found to contribute to endometrial regeneration and proliferation in humans [50] and rats [11], and the safety of ovarian in situ administration was also confirmed [51]. The secretomes of HucMSCs have also been proven to be significant functional modes for tissue regeneration, including small-sized extracellular vesicles [22] and apoptotic bodies [52]. In fact, MSCs are confirmed to be supporter cells for follicular growth in vitro [18], and their secretion of growth factors such as fibroblast growth factor (FGF) and epidermal growth factor (EGF) could be utilized in follicle growth [53]. Follicle IVC has been well established in protecting ovarian reserve for cancer patients who want fertility conservation [45]. However, aged follicles exhibit aberrant hormone secretion and diminished gamete health both during in vivo growth [54] and in vitro culture [20]. Few studies have focused on improving the development of aged follicles, and the IVC system of aged follicles needs more exploration and optimization. Therefore, we hypothesized that supplementation of HucMSC-EVs may be a safe and effective cell-free strategy to promote the development of aged follicles during IVC.

EVs can be taken up by various types of cells, including embryos, oocytes [55], granulosa cells [56], and even ovarian tissues [22]. Our findings further demonstrated uptake by preantral follicles and oocytes (Figs. 2A and 6A). We first confirmed the optimized dosage and coculture days of HucMSC-EVs on murine follicles, wherein we indeed found that 5 µg/mL HucMSC-EVs for 3 days increased the survival and growth of preantral follicles. The effect of MSCs improving follicular activation and early follicle development has been demonstrated [57]. Consistent with our study, the growth of small-aged follicles was notably increased after the stimulus with HucMSC-EVs. In addition, for large-sized follicles, HucMSC-EVs facilitated size expansion, elevated the antrum formation rate, and resulted in increased oocyte retrieval after the ovulation stimulus. MSC-derived EVs are proven to efficaciously enhance the proliferation level of GCs [56], which are essential for follicular development. We examined the cellular proliferation marker Ki67 and found it to be clearly upregulated in GCs, which is consistent with the upregulated genes related to “cell proliferation” and “negative regulation of apoptotic process” function in the RNA-seq analysis of GCs, which may be a potential underlying mechanism of HucMSC-EVs. The data also revealed some other potential mechanisms to improve the development of aged follicles. For example, the biological process of telomere organization enriched in the proteins of HucMSC-EVs and the negative regulation of telomere maintenance in the downregulated DEGs of RNA-seq analysis may be relevant to the phenotype of anti-ageing in the oocytes, which also need to be further elaborated.

Moreover, we assessed the expression levels of several oocyte-specific genes known to participate in oocyte development, including *Zar1*, *Zp3*, and *Nobox* [58]. *Gdf9* and *Bmp15* are factors secreted by oocytes that play important roles during follicular development [59]. Recent studies have shown that the anti-ageing gene *Sirt1* in oocytes has been demonstrated to maintain the homeostasis of oxidative stress and mitigate the age-related decline in oocyte quality [60], as well as inhibit apoptosis in GCs [61]. For GCs, *Fshr* is related to the response to FSH [62]; moreover, *Kitl* is related to the interaction and communication between oocytes and GCs [63], and BCL2 associated X (*Bax*) is an apoptosis-associated gene. These upregulated genes indicate the good quality of oocytes and improvement of granulosa cell functions. However, the results of the gene expression analysis of young follicles were not significantly different between the two groups after 7 days of culture (Supplementary Fig. 4), and we supposed that this may have been due to the near saturation of the developmental capacity of these young and healthy preantral follicles. A certain level of hormone secretion is a critical sign of follicle development; specifically, androgen is produced by theca cells, and the conversion to estrogen occurs via GCs in the preantral follicles [64]. Indeed, after the follicle matures and ovulates, GCs and theca cells are luteinized to form granulosa luteal cells and theca luteal cells, respectively. The corpus luteum is the main source of estrogen and progesterone after ovulation in vivo, and studies have shown that progesterone plays a very important role in the initiation and maintenance of pregnancy [65]. In our study, the E2 and P4 secretion levels were increased after supplementation with HucMSC-EVs, thus indicating the good quality of these aged preantral follicles.

During folliculogenesis, PI3K/Akt and mTOR signaling pathways were considerably emphasized in regulating primordial follicle activation [66] and secondary follicle growth [67]. PI3K/Akt pathway plays an obligatory role in the granulosa cell proliferation and differentiation in ovarian follicles at other stages [68]. Akt-stimulating drugs (PI3K activator) were proven to promote the growth of secondary follicles from humans and mice [69]. Specifically, mTORC1 signaling activation would elevate KITL expression in pre-granulosa cells, which can bind to KIT and activate PI3K/Akt signaling in oocytes and induce the phosphorylation of forkhead Box O3a (FOXO3a) and exportation from the nucleus, further leading to primordial follicle activation and oocyte growth [70]. Studies have shown that mTORC1 signaling would activate CREB, which is able to bind the promoter of the Kitl gene to enhance its expression and therefore influence the process of primordial follicle activation [71]. A recent study reported that hepatocyte growth factor (HGF) secreted by HucMSC was able to increase the expression of KITL in granulosa cells by HGF receptor (c-Met) and activate the PI3K/Akt pathway to promote primordial follicle activation [72], implying the potential associations of HucMSC-EVs in our study with these signaling pathways related to folliculogenesis. HDAC6 was verified to extend the murine reproductive lifespan [73] and dormant primordial follicle maintenance [38]. The inhibition of HDAC6 induced the phosphorylation of Akt in oocytes through upregulation of the mTOR-KITL pathway in pre-granulosa cells and resulted in primordial follicle activation [38]. Furthermore, SIRT1 has been proven to activate kinase Akt and regulate biological functions in a variety of tissues [74]. A previous study reported that SIRT1 could facilitate primordial follicle activation and promote oocyte growth via the direct modulation of the transcription of Akt1 and mTOR [39], thus implying the importance of epigenetic modification effect in the process of folliculogenesis. We indeed found a decrease of HDAC6 and an increase of SIRT1 expression, together with the temporary activation of the CREB/KITL/PI3K/Akt signaling pathway after coculture with HucMSC-EVs. However, the *Sirt1* mRNA expression level elevation after 7 days of coculture shown in Fig. 4G-H acted as the increased antioxidative capacity of aged follicles in the EVs group needed to be further elucidated. Additionally, studies have demonstrated the fact that several molecules or factors within HucMSC-EVs are capable of modulating PI3K/Akt sinaling pathway to affect different biological processes, such as microRNA (miR-455-3p [75], miR-21-5p [22], and miR-21 [76]), mRNA (human HGF mRNA [77]), which was in accordance with our enrichment results of HucMSC-EVs proteins (Fig. 1I). Apart from that, Wnt4 within HucMSC-EVs would promote wound healing via activation of Wnt/β-catenin signaling [78], which is another crucial pathway during follicle development. However, the exact functional molecule and the potential mechanism that facilitated the development of aged follicles and oocytes in our study needed further exploration.

Oocytes and other somatic cells of follicles are maintained in an active transcriptional and translational state during follicle growth, which can induce cell proliferation and differentiation, strengthen the communications between oocytes and GCs, and further improve the development of oocytes [79]. Interestingly, in our present study, the global cellular transcription level in the follicles (especially in the oocytes) after treatment with HucMSC-EVs was elevated (Fig. 5). Therefore, we performed RNA-seq analysis of these oocytes and GCs from aged follicles and found identical results. The GO enrichment analysis of the DEGs revealed that upregulated genes were relevant to “positive regulation of transcription from RNA polymerase II promoter” (Fig. 5G, J), which was also consistent with higher PSII and EU expression at the protein level. In addition, the proteasome of HucMSC-EVs was also investigated and was consistent with our conclusions. The function of “biosynthesis of amino acids” in the KEGG analysis showed a high correlation with translation activation, “DNA-templated transcription, initiation” in BP, “cell-cell junction” in CC, and “mRNA 5’-UTR binding” and “RNA binding” in MF of the GO analysis, which was also related to the initiation of cellular transcription (Fig. 1I-J). Consequently, we speculated that some proteins or non-coding RNAs encapsulated in HucMSC-EVs may activate the global transcription of cells after uptake by the follicles. Taken together, these consistent results suggest that the beneficial role of HucMSC-EVs on aged follicle growth may be attributed to the elevation in global transcription levels.

Despite the overall improvement in these aged follicles by coculture with HucMSC-EVs, the oocytes that were acquired after rEGF and hCG stimulation were still of low quality (Fig. 4C), which was possibly due to the fact that not all of the antral follicles on day 9 reached the preovulatory stage or because the oocytes had a low competence for maturity with advanced maternal age [20]. For a better understanding of the effect of HucMSC-EVs on aged oocytes, we used fully grown oocytes in the GV stage to evaluate the maturation process. We found that HucMSC-EVs improved the maturation rate of the aged oocytes, and these mature oocytes displayed fewer aberrant spindle morphologies and exhibited a higher translational level, which was a promising result for potential future applications (Fig. 6). SIRT1 has been proven to be a key metabolic sensor for oocyte homeostasis and plays an important role in oocyte maturation, which has been verified to be decreased among aged oocytes [61] (Supplementary Fig. 1I-J). In some studies, activation of SIRT1 during the IVM process of murine oocytes improved the oocyte and embryo developmental capacity [80]. We found that oocytes in the EVs groups showed more potential antioxidative competence with higher levels of SIRT1. However, the potential mechanism of SIRT1 activation in aged follicles and oocytes needs to be further investigated and illustrated.

As shown in Supplementary Fig. 1, follicles from aged mice had a lower capacity to form healthy gametes compared with those follicles from juvenile mice, which was possibly due to the early influences from the impaired ovarian microenvironment from aged females. A previous study demonstrated that aged murine oocytes could improve their reproductive quality when removed from preovulatory follicles and matured in vitro [81], thus implying the hypothesis that the aged follicular microenvironment can elicit a detrimental nuclear and cytoplasmic effect on the oocytes [54]. In our opinion, the combination of the IVA, IVC, and IVM culture systems would benefit patients with ovarian ageing with more gametes of better quality to achieve further successful in vitro fertilization. HucMSC-EVs serve as a type of cell-free regenerative medicine in the lack of immunogenicity and can be safely supplemented into the IVC and IVM culture medium. Our study specifically displayed the process of increasing the in vitro developmental capacity of aged follicles from the early preantral stage, as well as aged oocytes by HucMSC-EVs, thus providing the preclinical experimental foundation and proposing a brand-new insight for the improvement of female fecundity.

## Conclusion

In summary, our findings demonstrated that HucMSC-EVs were capable of facilitating the development of aged preantral follicles and improving the quality of these follicles, which was partially accomplished by elevating the global cellular transcriptional level. In addition, HucMSC-EVs also promoted the maturation process of aged murine oocytes. These results powerfully confirmed the functional effect of mesenchymal stem cell-derived EVs on ovarian follicle growth and provide new insights into stem cell therapy for the reproductive and fertility preservation of advanced maternal age.

## Supplementary Information

Below is the link to the electronic supplementary material.

Supplementary Fig. 1 Comparisons of follicles and oocytes obtained from the young and aged mice. **A** Immunofluorescence of Ki67 in preantral follicles in young and aged ovary tissues. Red represents Ki67. Blue represents Hoechst 33,342. Scale bar = 25 μm. **B** Quantitative analysis of the survival rate among young follicles (n = 78) and aged follicles (n = 80). **C** The antrum formation rate of large-sized survived young follicles (n = 26) was compared to aged ones (n = 27). **D** Phase-contrast images of oocytes ovulated from young and aged follicles after in vitro ovulation stimulation. The red star indicates the oocytes that reached the MII stage. Scale bar = 100 μm. **E** The oocyte retrieval rate of the total survived follicles was assessed in the young group (n = 84) and the aged group (n = 46). **F** Representative images of oocytes after in vivo ovulation (IVO) stimulation. Scale bar = 100 μm. **G** Quantitative analysis of the number of total ovulated oocytes per mouse among young (n = 3) and aged (n = 4) groups. **H** The rate of abnormal morphology oocytes in the aged group (n = 105) compared to the young group (n = 115). **I** Representative confocal images of SIRT1 in the two groups. Scale bar = 25 μm. **J** Statistical analysis of the fluorescence intensity of SIRT1 in young (n = 10) and aged (n = 9) oocytes. **K** Representative images of young and aged oocyte maturation in vitro. Scale bar = 50 μm. The red arrowhead indicates the oocytes that failed to extrude a polar body or experience degeneration. **L** Quantitative measurement of the IVM rate in young (n = 118) and aged (n = 108) oocytes. ns: not significant, **P < 0.01, ***P < 0.001.(PNG 4.09 MB)

High Resolution Image (TIF 10.8 MB)

Supplementary Fig. 2 Potential mechanism of HucMSC-EVs promoting the development of aged follicles. Western blot analysis of the CREB/KITL/PI3K/Akt signaling pathway in **A** small-sized and **B** large-sized aged follicles with or without HucMSC-EVs after 4-8-16-24 h coculture, respectively. **C** Representative immunofluorescence of HDAC6 in the control and EVs groups. Scale bar = 25 μm. **D** Statistical analysis of the fluorescence intensity of HDAC6 in follicles with or without HucMSC-EVs. **E** Western blot analysis of the SIRT1 expression of aged follicles with or without HucMSC-EVs after 8-16-24 h of coculture, respectively. The expression of β-ACTIN was used as the internal control. ***P < 0.001. (PNG 1.19 MB)

High Resolution Image (TIF 5.12 MB)

Supplementary Fig. 3 Heatmap plot of all differentially expressed genes (DEGs) in **A** oocyte samples and **B** GC samples showed clustering of the two groups. (PNG 111 KB)

High Resolution Image (TIF 774 KB)

Supplementary Fig. 4 qPCR analysis of relative mRNA levels in **A** GCs and **B** oocytes separated mechanically from young murine follicles on culture day 7. *P < 0.05. (PNG 64.9 KB)

High Resolution Image (TIF 508 KB)

Supplementary Table 1 Primers used in the study for qPCR analysis. (XLSX 10.4 KB)

Supplementary Table 2 List of proteins identified by proteomic analysis of HucMSC-EVs. (XLSX 132 KB)

Supplementary Table 3 Gene expression levels of 16 samples by FPKM in the RNA-seq analysis. (XLSX 3.93 MB)

Supplementary Table 4 List of used antibodies in the study. (XLSX 10.1 KB)

## Data Availability

The materials and analysed datasets used in the current study are available from the corresponding author upon reasonable request.

## Abbreviations

IVC: In vitro culture
IVM: In vitro maturation
MSCs: Mesenchymal stem cells
HucMSCs: Human umbilical cord-derived mesenchymal stem cells
EVs: Extracellular vesicles
HucMSC-EVs: Extracellular vesicles derived from HucMSCs
IVA: In vitro activation
POI: Premature ovarian insufficiency
DOR: Diminished ovarian reserve
MII: Metaphase II
IVO: In vivo ovulated
GV: Germinal vesicle
hCG: Human chorionic gonadotrophin
rEGF: Recombinant human epidermal growth factor
COC: Cumulus-oocyte complex
mRNA: Messenger RNA
qPCR: Real-time quantitative polymerase chain reaction
PSII: RNA Polymerase II
E2: Oestrogen
P4: Progesterone
GCs: Granulosa cells
LC-MS/MS: Liquid chromatography-tandem MS
RNA-seq: RNA sequencing
DEGs: Differentially expressed genes
GO: Gene Ontology
KEGG: Kyoto Encyclopedia of Genes and Genomes
FPKM: Fragments per kilobase of transcripts per million mapped reads
