# Supplementary figures and images for: HucMSC-EVs Facilitate In Vitro Development of Maternally Aged Preantral Follicles and Oocytes
Source: Stem Cell Rev Rep. 2023 Mar 2;19(5):1427–48. doi: 10.1007/s12015-022-10495-w (PMC10366269; doi:10.1007/s12015-022-10495-w)

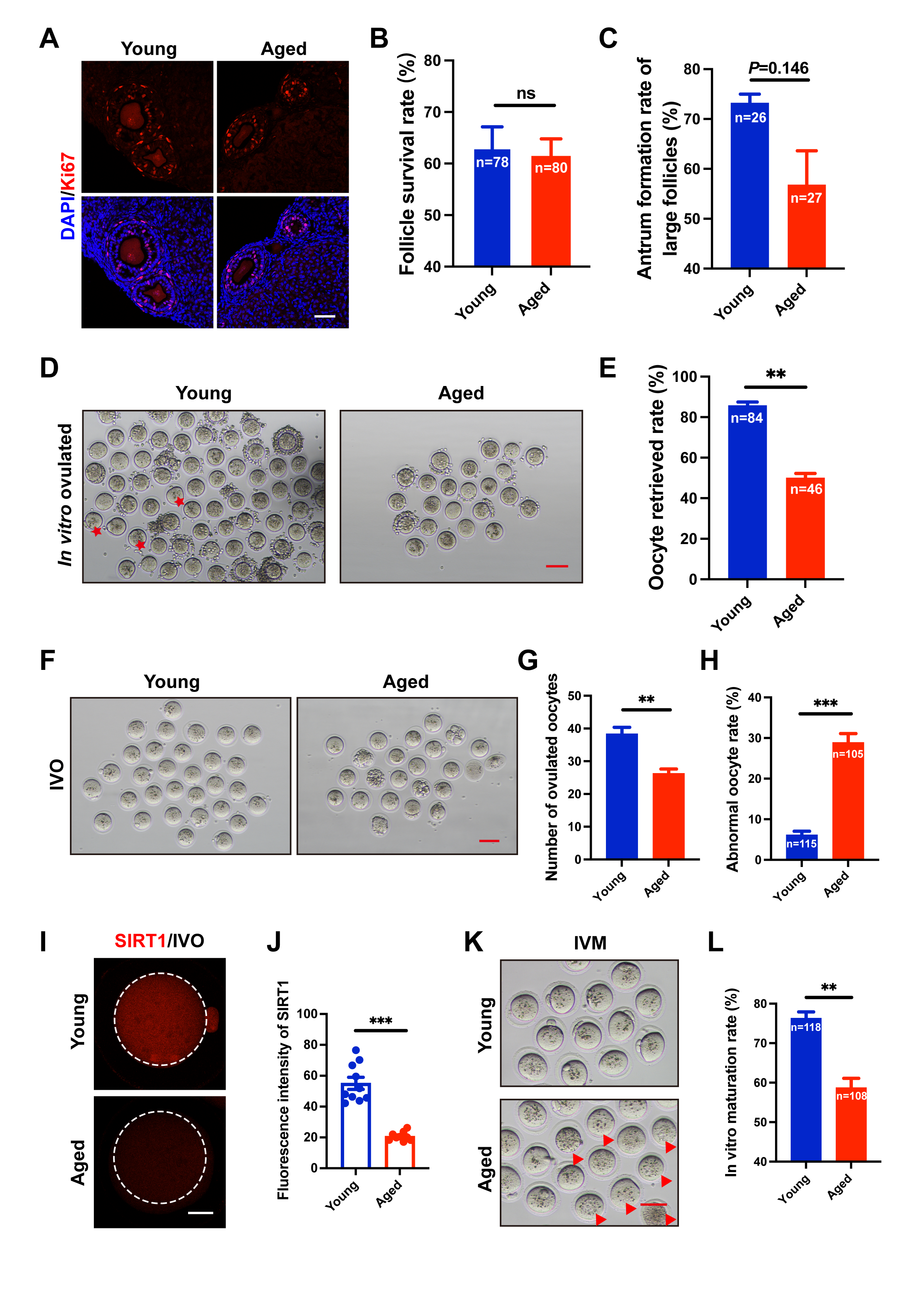

Supplement: Supplementary file 1 — Comparisons of follicles and oocytes obtained from the young and aged mice. A Immunofluorescence of Ki67 in preantral follicles in young and aged ovary tissues. Red represents Ki67. Blue represents Hoechst 33,342. Scale bar = 25 μm. B Quantitative analysis of the survival rate among young follicles (n = 78) and aged follicles (n = 80). C The antrum formation rate of large-sized survived young follicles (n = 26) was compared to aged ones (n = 27). D Phase-contrast images of oocytes ovulated from young and aged follicles after in vitro ovulation stimulation. The red star indicates the oocytes that reached the MII stage. Scale bar = 100 μm. E The oocyte retrieval rate of the total survived follicles was assessed in the young group (n = 84) and the aged group (n = 46). F Representative images of oocytes after in vivo ovulation (IVO) stimulation. Scale bar = 100 μm. G Quantitative analysis of the number of total ovulated oocytes per mouse among young (n = 3) and aged (n = 4) groups. H The rate of abnormal morphology oocytes in the aged group (n = 105) compared to the young group (n = 115). I Representative confocal images of SIRT1 in the two groups. Scale bar = 25 μm. J Statistical analysis of the fluorescence intensity of SIRT1 in young (n = 10) and aged (n = 9) oocytes. K Representative images of young and aged oocyte maturation in vitro. Scale bar = 50 μm. The red arrowhead indicates the oocytes that failed to extrude a polar body or experience degeneration. L Quantitative measurement of the IVM rate in young (n = 118) and aged (n = 108) oocytes. ns: not significant, **P < 0.01, ***P < 0.001.(PNG 4.09 MB) [file 12015_2022_10495_Fig7_ESM.png]

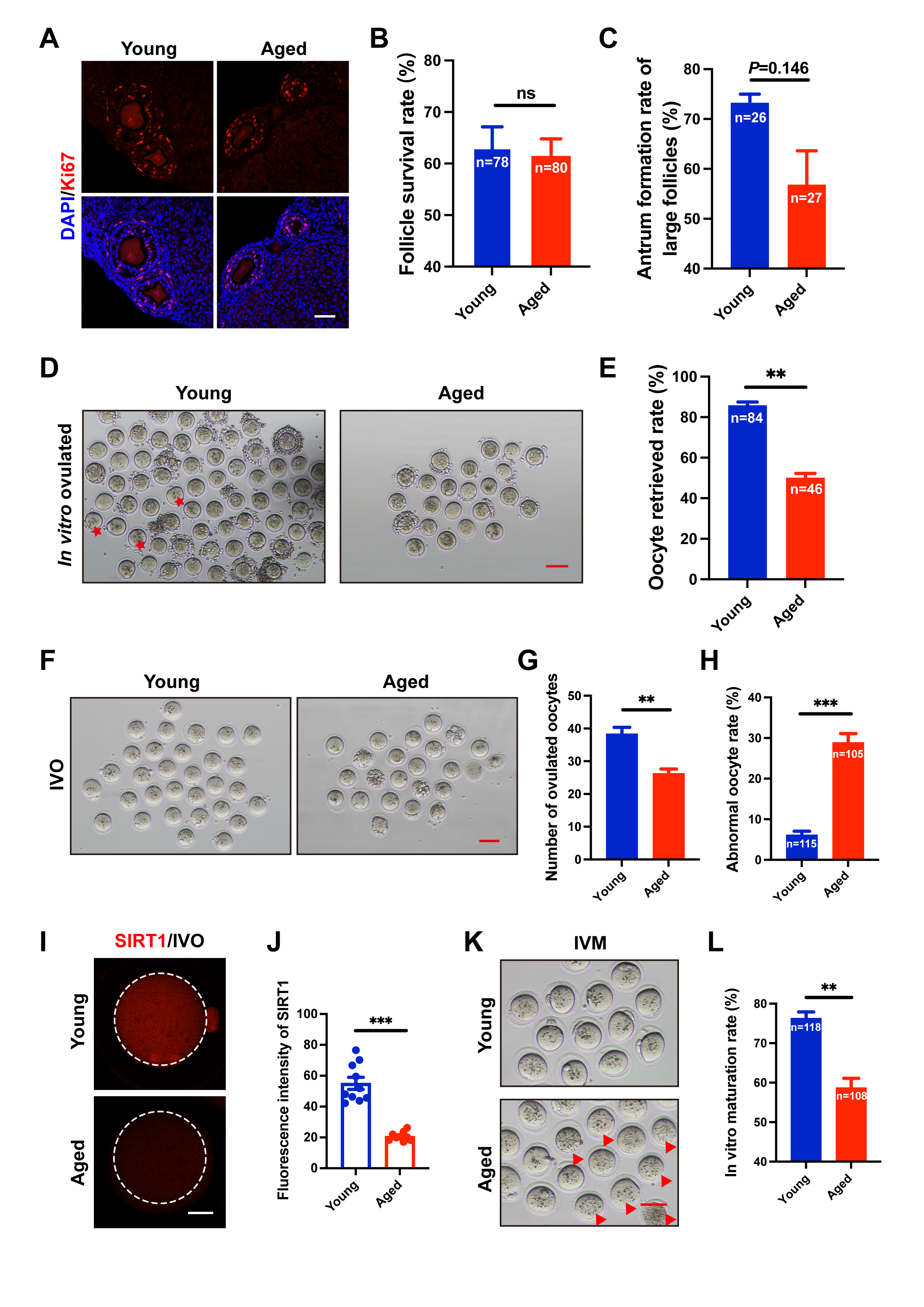

Supplement: Supplementary file 2 — High Resolution Image (TIF 10.8 MB) [file 12015_2022_10495_MOESM1_ESM.tif]

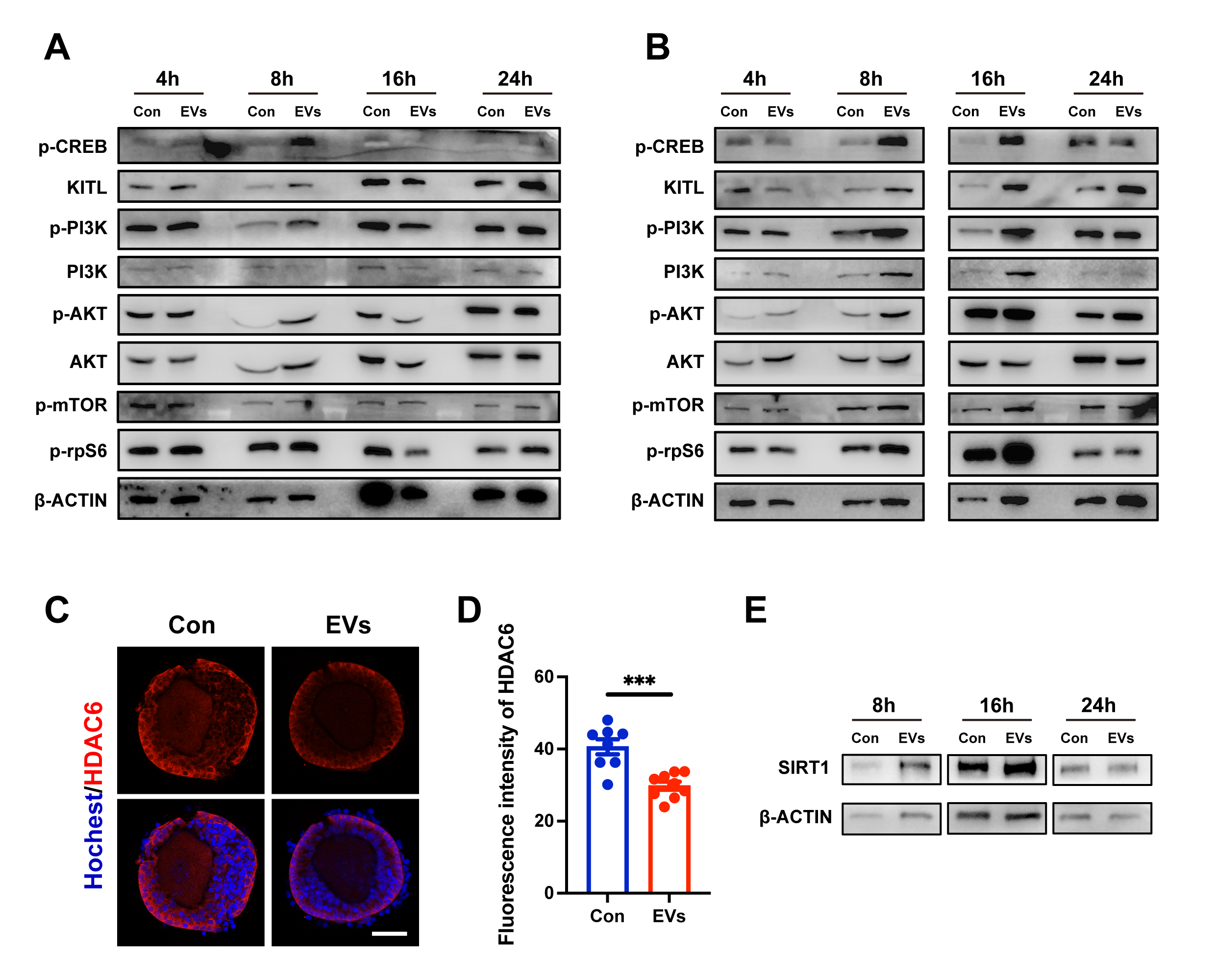

Supplement: Supplementary file 3 — Potential mechanism of HucMSC-EVs promoting the development of aged follicles. Western blot analysis of the CREB/KITL/PI3K/Akt signaling pathway in A small-sized and B large-sized aged follicles with or without HucMSC-EVs after 4-8-16-24 h coculture, respectively. C Representative immunofluorescence of HDAC6 in the control and EVs groups. Scale bar = 25 μm. D Statistical analysis of the fluorescence intensity of HDAC6 in follicles with or without HucMSC-EVs. E Western blot analysis of the SIRT1 expression of aged follicles with or without HucMSC-EVs after 8-16-24 h of coculture, respectively. The expression of β-ACTIN was used as the internal control. ***P < 0.001. (PNG 1.19 MB) [file 12015_2022_10495_Fig8_ESM.png]

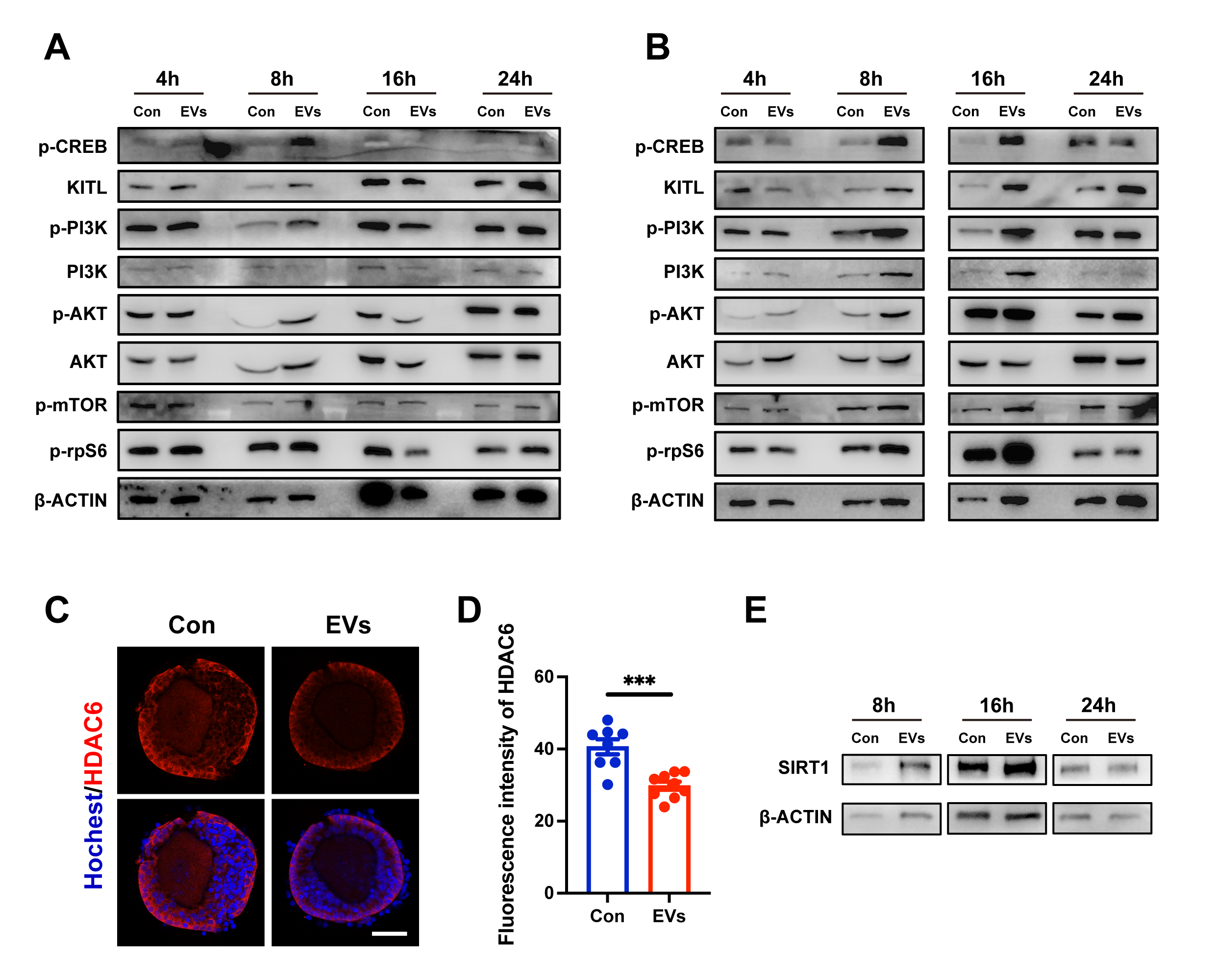

Supplement: Supplementary file 4 — High Resolution Image (TIF 5.12 MB) [file 12015_2022_10495_MOESM2_ESM.tif]

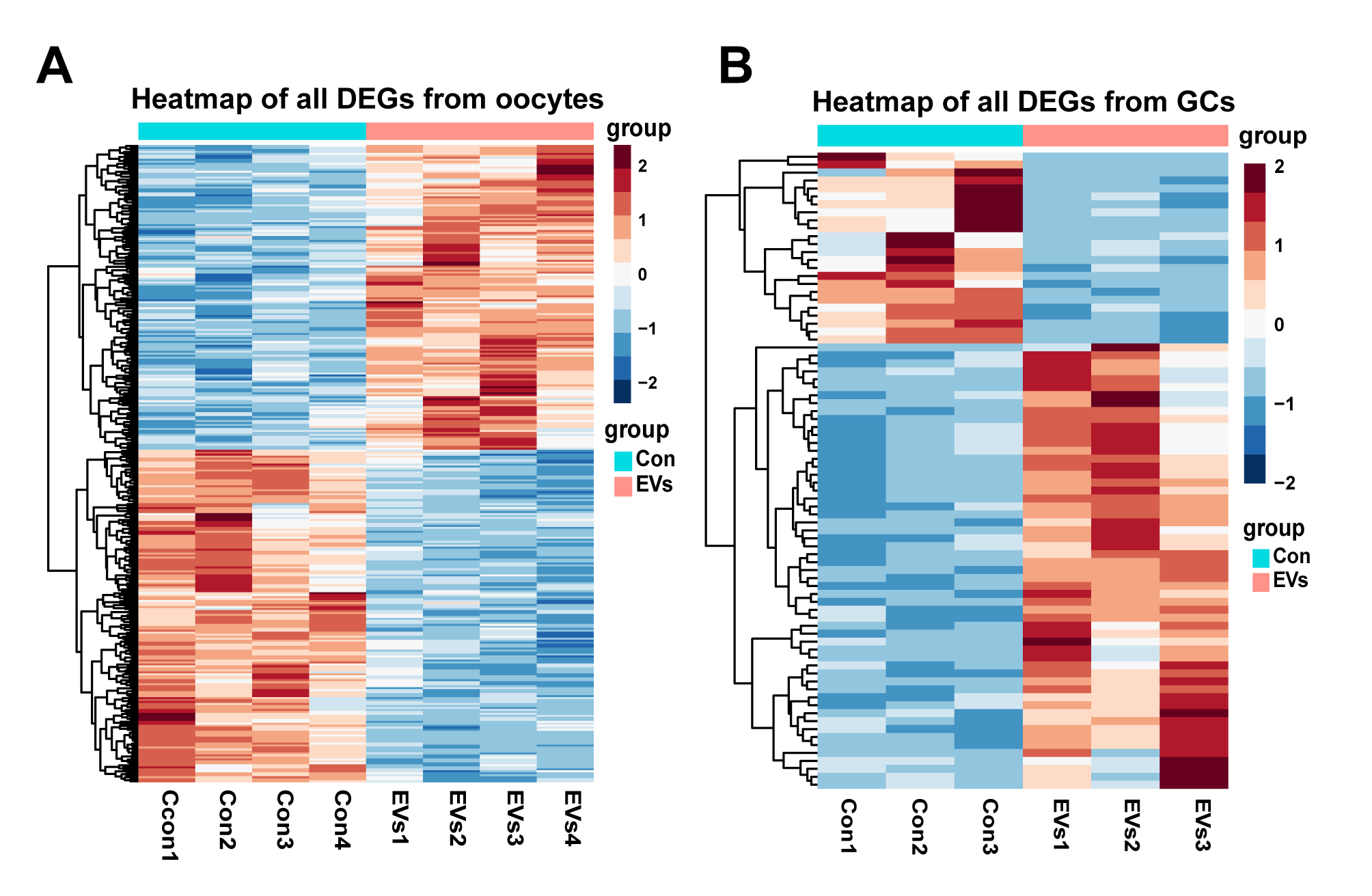

Supplement: Supplementary file 5 — Heatmap plot of all differentially expressed genes (DEGs) in A oocyte samples and B GC samples showed clustering of the two groups. (PNG 111 KB) [file 12015_2022_10495_Fig9_ESM.png]

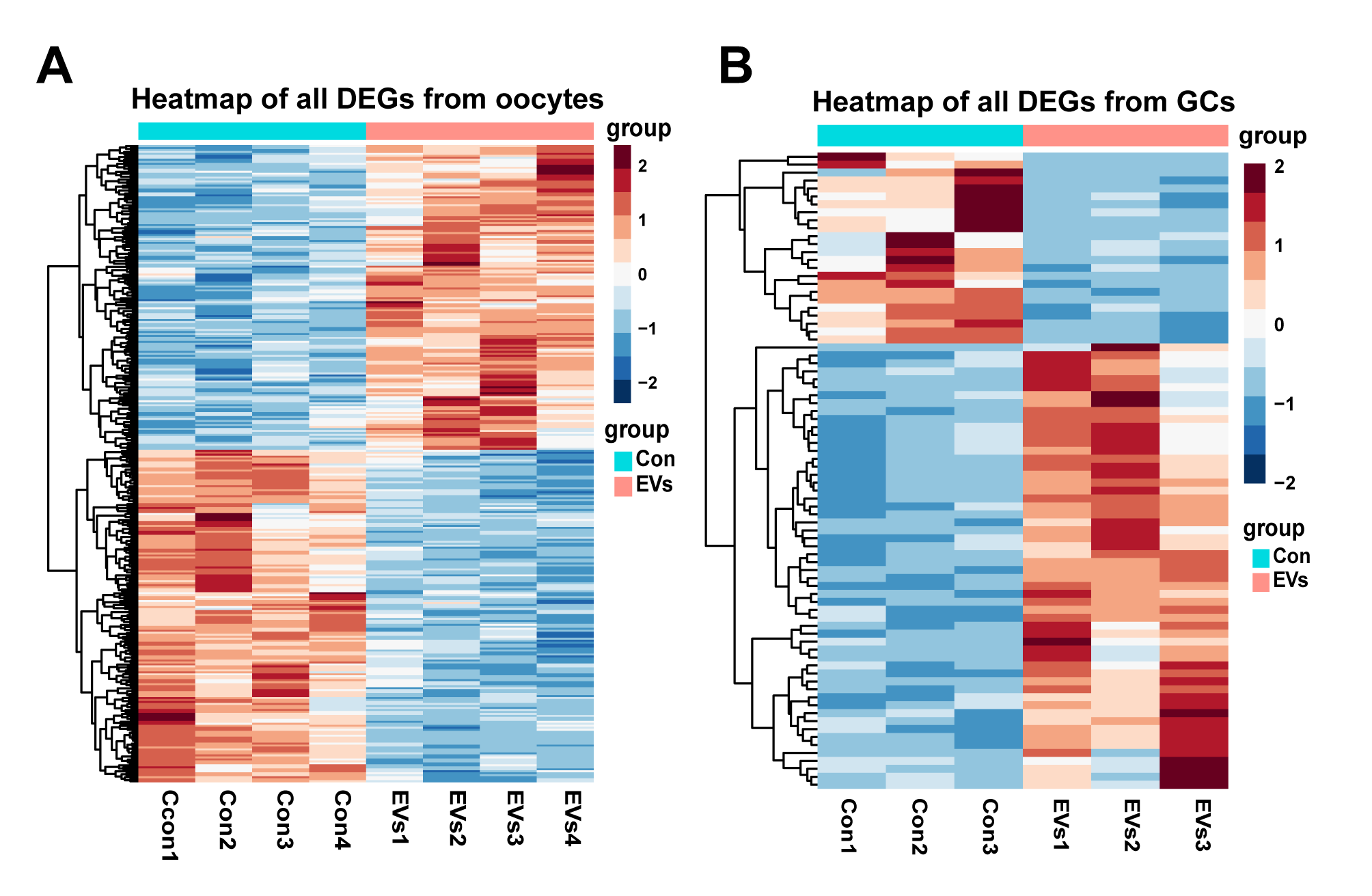

Supplement: Supplementary file 6 — High Resolution Image (TIF 774 KB) [file 12015_2022_10495_MOESM3_ESM.tif]

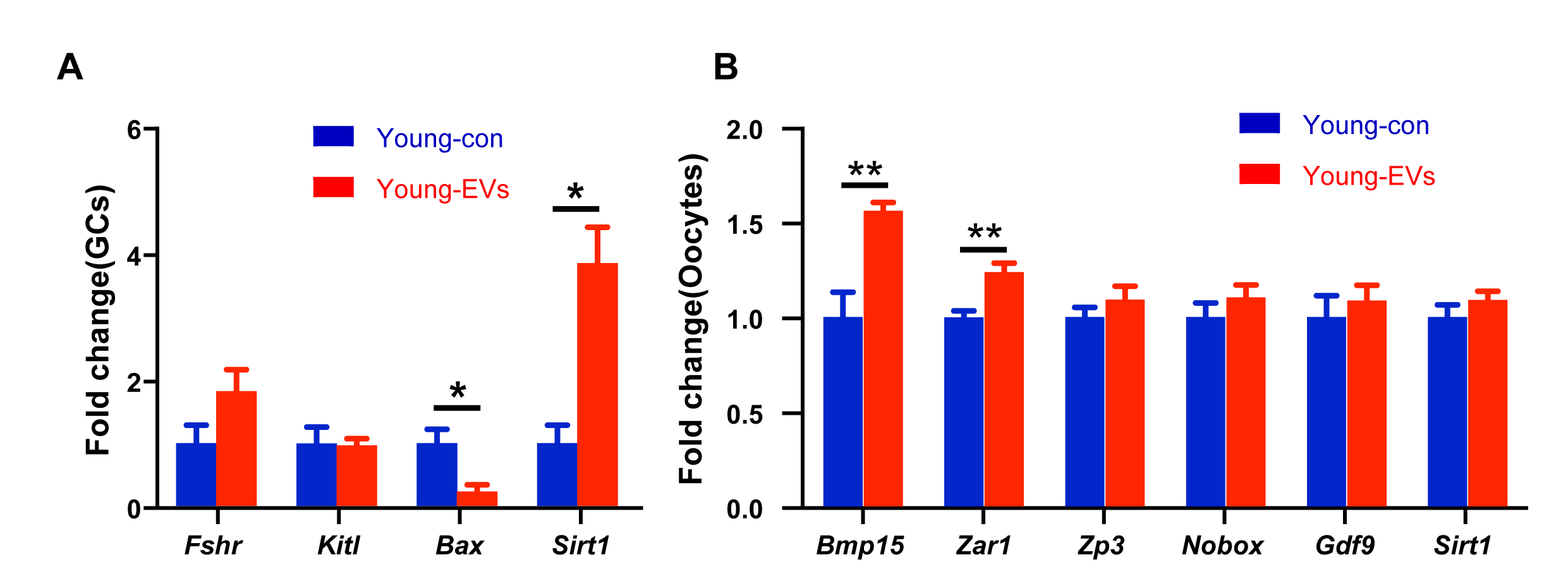

Supplement: Supplementary file 7 — qPCR analysis of relative mRNA levels in A GCs and B oocytes separated mechanically from young murine follicles on culture day 7. *P < 0.05. (PNG 64.9 KB) [file 12015_2022_10495_Fig10_ESM.png]

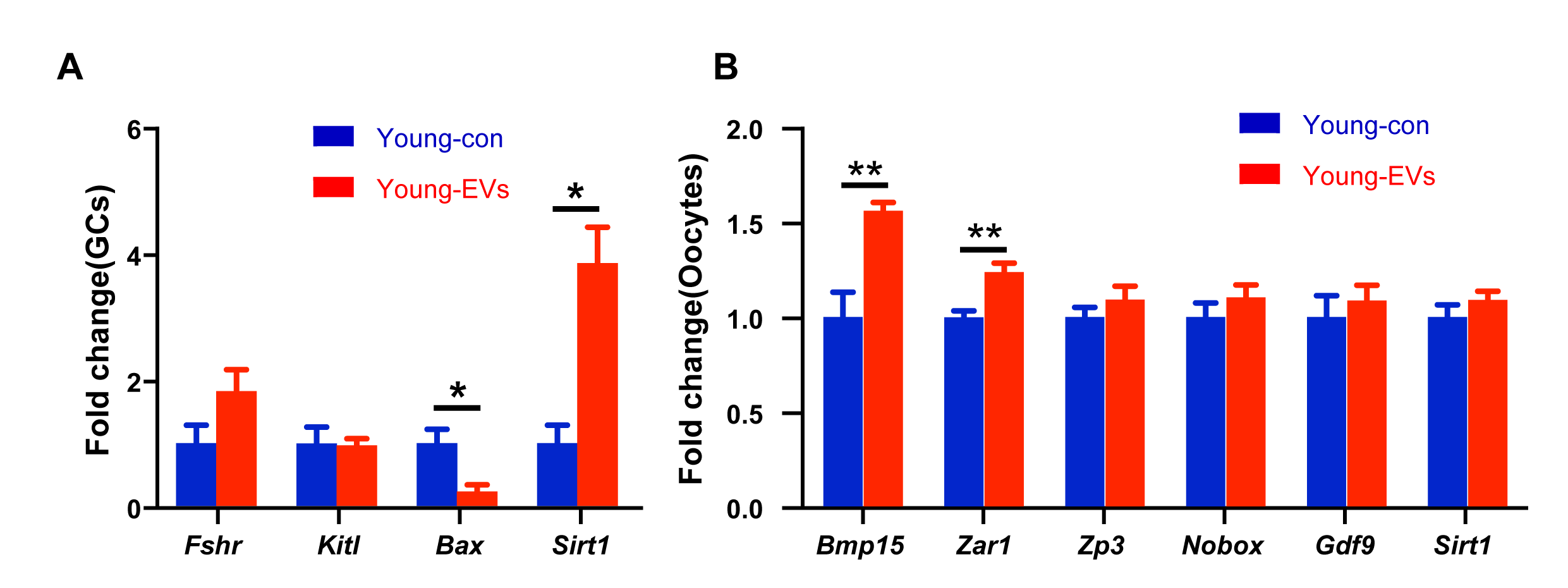

Supplement: Supplementary file 8 — High Resolution Image (TIF 508 KB) [file 12015_2022_10495_MOESM4_ESM.tif]
